# miR-4711-5p regulates cancer stemness and cell cycle progression via KLF5, MDM2 and TFDP1 in colon cancer cells

**DOI:** 10.1038/s41416-020-0758-1

**Published:** 2020-02-18

**Authors:** Yoshihiro Morimoto, Tsunekazu Mizushima, Xin Wu, Daisuke Okuzaki, Yuhki Yokoyama, Akira Inoue, Tsuyoshi Hata, Haruka Hirose, Yamin Qian, Jiaqi Wang, Norikatsu Miyoshi, Hidekazu Takahashi, Naotsugu Haraguchi, Chu Matsuda, Yuichiro Doki, Masaki Mori, Hirofumi Yamamoto

**Affiliations:** 1 Department of Surgery, Gastroenterological Surgery, Graduate School of Medicine, Osaka University, Yamadaoka 2-2, Suita city, Osaka 565-0871 Japan; 2 Department of Molecular Pathology, Division of Health Sciences, Graduate School of Medicine, Osaka University, Yamadaoka 1-7, Suita city, Osaka 565-0871 Japan; 3 Genome Information Research Centre, Research Institute for Microbial Diseases, Osaka University, Yamadaoka 3-1, Suita city, Osaka 565-0871 Japan; 4 Department of Surgery, Graduate School of Medical Sciences, Kyushu University, 3-1-1, Maidashi, Higashi-ku, Fukuoka city, Fukuoka 812-8582 Japan

**Keywords:** Drug development, Cancer stem cells

## Abstract

**Background:**

It is important to establish cancer stem cell (CSC)-targeted therapies to eradicate cancer. As it is a CSC marker, we focused on *Kruppel-like factor* 5 (*KLF5*) in this study.

**Methods:**

We searched for candidate microRNAs (miRNAs) that inhibited *KLF5* expression by in silico analyses and screened them in colon cancer cell lines.

**Results:**

We identified one promising miRNA, miR-4711-5p, that downregulated KLF5 expression by direct binding. This miRNA suppressed cell proliferation, migration and invasion ability, as well as stemness, including decreased stem cell marker expression, reactive oxygen species activity and sphere formation ability. MiR-4711-5p inhibited the growth of DLD-1 xenografts in nude mice with no adverse effects. We found that miR-4711-5p provoked G1 arrest, which could be attributed to direct binding of miR-4711-5p to *TFDP1* (a heterodimeric partner of the E2F family). Our findings also suggested that direct binding of miR-4711-5p to *MDM2* could upregulate wild-type p53, leading to strong induction of apoptosis. Finally, we found that miR-4711-5p had a potent tumour-suppressive effect compared with a putative anti-oncomiR, miR-34a, in tumour cell cultures derived from five patients with colorectal cancer.

**Conclusions:**

Our data suggest that miR-4711-5p could be a promising target for CSC therapy.

## Background

Colorectal cancer (CRC) is the third most commonly diagnosed cancer in males and the second most commonly diagnosed cancer in females worldwide.^1^ Great advancements have been made in CRC treatments, including surgery, radiochemotherapy and the development of monoclonal antibodies against vascular endothelial growth factor (VEGF) and epidermal growth factor receptor (EGFR). However, the 5-year survival remains less than 65%.^2,3^

Current evidence suggests that cancer tissues arise from a small sub-population of cells, termed cancer stem cells (CSCs), which reside in a tumour and possess the capacities to self-renew and to generate the heterogeneous lineages of cancer cells that comprise the tumour body.^4,5^ Because CSCs are considered to be responsible for therapeutic tolerance and disease recurrence,^6,7^ it is important to establish CSC-targeted therapies to eradicate cancer.

Kruppel-like factor 5 (KLF5) is a zinc-finger transcription factor of the KLF family. KLF family proteins share homology in their carboxyl-terminal zinc-finger domains, which allow KLFs to bind GC-rich sites in the promoter and enhancer regions of the specific genes they regulate.^8^ KLF family proteins play various roles in the homeostasis and pathogenesis of the cardiovascular and renal systems and in lipid metabolism.^9^ As it is a CSC marker, in this study, we focused on KLF5 because KLF5 has the potential to generate induced pluripotent stem cells and controls the stemness of embryonic stem cells.^10,11^ Furthermore, KLF5 is expressed at higher levels in the crypts, where stem cells reside, than in other areas,^12^ and it is abundantly expressed in cervical cancer and gastrointestinal tract cancers such as colorectal cancer and gastric cancer (Supplementary Fig. S1). KLF5 acts as a core regulator of intestinal oncogenesis, as demonstrated in a genetically manipulated mouse model that exhibits simultaneous oncogenic activation of Wnt/β-catenin signalling and KLF5 deletion, specifically in Lgr5^+^ intestinal stem cells.^13^ Overall, these findings suggest that KLF5 may be an appropriate target in a strategy to eradicate stem-like cells in CRC.

MicroRNAs (miRNAs) are single-stranded noncoding RNAs comprising 20–22 nucleotides. MiRNAs bind to complementary sequences, mainly in the 3ʹ-untranslated regions (3ʹ-UTRs) of target mRNAs and can cause translational repression or facilitate target mRNA cleavage.^14,15^ MiRNA-based therapy is expected to be a successful next-generation treatment, since miRNAs are involved in various biological processes and can simultaneously regulate the expression of multiple target genes.^16^

In the present study, we searched for miRNAs that negatively regulate KLF5 expression. In silico analyses revealed one promising miRNA: miR-4711-5p. This miRNA suppressed CSC properties, possessed various anti-tumour effects against CRC cell lines and CRC cell cultures derived from clinical CRC samples through direct inhibition of KLF5 and MDM2, and provoked G1 arrest via direct inhibition of TFDP1, which forms a heterodimer with E2F1. Moreover, miR-4711-5p was proven to be safe when administered to mice and thus may have the potential to be utilised for CSC-targeted therapy against CRC.

## Methods

### Cell lines and cell culture

We obtained the human colorectal cancer cell lines DLD-1, HCT116 and HT29 from the American Type Culture Collection in 2001. All cells were cultured in Dulbecco’s modified Eagle’s medium (DMEM) containing 10% foetal bovine serum (FBS), 100 U/mL penicillin and 100 mg/mL streptomycin. Cells were cultured at 37 °C in 5% CO_2_. The cell lines were authenticated by morphological inspection, short tandem repeat profiling and mycoplasma testing. Mycoplasma testing was also performed by the authors in 2015.

### Transient transfection of miRNA/plasmid

From Gene Design Inc. (Osaka, Japan), we obtained the following oligonucleotides: mimic-miR-4711-5p, mimic-miR-21-5p, mimic-miR-34a-5p and mimic-miR-negative control. Mimic-miR-152-5p, mimic-miR-153-3p and mimic-miR-448-3p were purchased from Bioneer Corporation (Daejeon, Korea). The sense and antisense sequences of each miRNA are described in Supplementary Table S1A. Cells were transfected with the miRNAs at concentrations of 20–33 nmol/L using Lipofectamine 2000 (Invitrogen, Carlsbad, CA, USA) according to the manufacturer’s protocol.

### RNA isolation

Total mRNA or miRNA was extracted using the miRNeasy Kit (Qiagen, Hilden, Germany). Total RNA concentration and purity were assessed using a NanoDrop ND-2000 spectrophotometer (Thermo Fisher Scientific, Waltham, MA, USA).

### Real-time quantitative PCR analysis of mRNA expression

From 1.0 μg total RNA, complementary DNA was synthesised using the High Capacity cDNA Reverse Transcription Kit (Applied Biosystems, Waltham, MA, USA). PCR was performed with the LightCycler 480 Real-Time PCR system (Roche Diagnostics, Basel, Switzerland), and the specific primers used are summarised in Supplementary Table S1B. Each gene expression value was normalised to the mRNA expression level of GAPDH.

### miRNA expression

miRNA expression was measured using TaqMan miRNA Assays (Applied Biosystems). The reverse transcription reaction was performed with the TaqMan MicroRNA RT Kit (Applied Biosystems) according to the manufacturer’s protocol. Quantitative real-time PCR was performed with the 7900HT Sequence Detection System (Applied Biosystems). Amplification data were normalised to endogenous RNU6B expression. Relative expression was quantified with the ΔΔCt method.

### Cell proliferation assay

Cells were seeded at a density of 3000–5000 cells per well in 96-well plates and cultured for 24–72 h. Cell counting was performed using the Countess Automated Cell Counter (Thermo Fisher Scientific). Cellular proliferation was evaluated with the Cell Counting Kit-8 (Dojindo Molecular Technologies, Inc. Kumamoto, Japan).

### Invasion assay

Cells were seeded in a 6-well plate at a density of 1 × 10^5^ cells per well, incubated overnight, and then transfected with miRNAs. At 24 h after transfection, the cells were reseeded in BD BioCoat Matrigel Invasion Chambers (BD Biosciences, Franklin Lakes, New Jersey, USA). Invading cells were stained with haematoxylin and counted at 48 h after reseeding.

### Wound healing assay

Cells were seeded at a density of 4 × 10^5^ cells per well in an ibidi 2-well Culture Insert (ibidi, Munich, Germany) set in a 24-well plate. At 24 h after seeding, the inserts were removed to create wounds. The wound area was calculated at 0, 24, 48 and 72 h after insert removal.

### Apoptosis assay

Apoptosis was assessed by flow cytometry analyses for Annexin V and PI. At 48 h after transfection, apoptotic cells were stained with the Alexa Fluor 488 Annexin V/Dead Cell Apoptosis Kit (Thermo Fisher Scientific) and assessed by flow cytometry using the SH800Z Cell Sorter (Sony Biotechnology Inc. San Jose, CA, USA). For the TUNEL (TdT-mediated dUTP nick end labelling) assay, formalin-fixed, paraffin-embedded tumours were prepared and sectioned at 4 μm. They were deparaffinised with xylene and then rehydrated in graded alcohols. Apoptotic DNA fragmentation within the cell was visualised with an In Situ Cell Death Detection Kit, Fluorescein (Roche Diagnostics), according to the manufacturer’s protocol. Nuclei were stained using Hoechst 33342 (Thermo Fisher Scientific). The localised green fluorescence of apoptotic cells was analysed with a BZ-X analyser (KEYENCE, Osaka, Japan).

### pmirGLO plasmid vector for luciferase reporter assay

RT-PCR was performed to amplify parts of the 3′-UTRs of KLF5, MDM2 and TFDP1 mRNA. The primer sequences were as follows: insert 1 of KLF5 (part of the 3ʹ-UTR of KLF5 mRNA; amplified product size, 250 bp), forward 5ʹ-GCTCGCTAGCCTCGATGTTGAAAACTTAAGGAAGCAAA-3ʹ, reverse 5ʹ-ATGCCTGCAGGTCGAATTGGGCAGCAAAGAGATG-3ʹ; insert 2 of KLF5 (another part of the 3ʹ-UTR of KLF5 mRNA; amplified product size, 205 bp), forward 5ʹ-GCTCGCTAGCCTCGAAAGCTAAACGCAATGTCATTTTT-3ʹ, reverse 5ʹ-ATGCCTGCAGGTCGACTGTGTCTCACTGTGTATTACATGC-5ʹ; insert of MDM2 (part of the 3ʹ-UTR of MDM2 mRNA; amplified product size, 469 bp): forward 5ʹ-GCTCGCTAGCCTCGAGACCCATCTACCCTGACCACA-3ʹ, reverse 5ʹ-ATGCCTGCAGGTCGACGAAACCTCTGCCATGTCTGC-5ʹ; and insert of TFDP1 (part of the 3ʹ-UTR of TFDP1 mRNA; amplified product size, 335 bp): forward 5ʹ-GCTCGCTAGCCTCGAGGGGTTTTCTGTTTCCTTTTGG-3ʹ, reverse 5ʹ-ATGCCTGCAGGTCGACCCATCCACAACAGGAGGTGT-3ʹ. The amplified products were subcloned and ligated into the multi-cloning site between Sal I and Xho I in the pmirGLO Dual-Luciferase miRNA Target Expression Vector (Promega) using the In-Fusion HD Cloning Kit (Clontech, Mountain View, CA, USA). The vectors with mismatched 3’-UTR sequences were constructed using the QuikChange Site-Directed Mutagenesis Kit (Agilent Technologies, Santa Clara, CA, USA) according to the manufacturer’s protocol. The sequences of inserts and vectors were confirmed by Sanger sequencing.

### Luciferase reporter assay

Cells were seeded in 96-well plates at a density of 5000 cells per well and were co-transfected with 50 ng pmirGLO plasmid vectors containing each insert and either miR-negative control (5 pmol) or miR-4711-5p (5 pmol). At 24 h after transfection, cells were assayed for both firefly and Renilla luciferase using the Dual-Luciferase Reporter Assay System (Promega). All experiments were conducted in triplicate.

### Western blot analysis

Western blot analyses were performed as previously described.^17^ Whole cells were lysed in RIPA buffer containing phosphatase inhibitor and protease inhibitor cocktail. Antibodies against proteins are listed in Supplementary Table S2.

### Cell cycle assay

For the cell cycle assay, cells were starved and incubated for 2 days without FBS to induce starvation. MiR-negative control or miR-4711-5p was transfected 36 or 24 h before the end of starvation for DLD-1 or HCT116 cells, respectively. At the end of starvation, FBS was added. Cells were fixed in 70% ethanol for 30 min and stored at 4 °C until use. Cells were washed twice with PBS and incubated with RNase (Sigma Aldrich, St. Louis, Missouri, USA) for 20 min at 37 °C and treated with PI (Dojindo Molecular Technologies) for 20 min on ice. The cell suspension was then filtered through a 60 μm Spectra mesh filter and analysed by flow cytometry using the SH800Z Cell Sorter (Sony Biotechnology Inc.). The percentage of cells in different phases of the cell cycle was determined with the FlowJo computer programme version 10.5.3 (BD Biosciences).

### ROS activity assay

Reactive oxygen species (ROS) activity was assessed by flow cytometry analyses. At 24 h after transfection, cells with high ROS activity were stained using the CellROX™ Deep Red Flow Cytometry Assay Kit (Invitrogen) and assessed by flow cytometry using the SH800Z Cell Sorter (Sony Biotechnology Inc.).

### Sphere formation assay

At 24 h after transfection with miR-negative control or miR-4711-5p, single cells were seeded in 96-well ultralow attachment plates (Corning Inc. Corning, NY, USA) at a density of 1000 cells per well. These cells were cultured in DMEM/F-12 serum-free medium supplemented with 20 ng/mL epithelial growth factor, 10 ng/mL fibroblast growth factor-2, and 100 μg/mL penicillin G at 37 °C in a humidified atmosphere of 95% air and 5% CO_2_. At 1 week after seeding, we counted the number of spheres ≥40 µm.

### RNA sequencing

We conducted RNA sequencing as previously described.^18^ The library was prepared using a TruSeq Stranded mRNA Sample Prep Kit (Illumina, San Diego, CA, USA). Sequencing was performed using the Illumina HiSeq 2500 platform in 75-base single-end mode. Illumina Casava 1.8.2 software was used for base calling, and the sequenced reads were mapped to human reference genome sequences (hg19) using TopHat version 2.0.13 combined with Bowtie2 version 2.2.3 and SAMtools version 0.1.19. We calculated the fragments per kilobase of exon per million mapped fragments (FPKMs) using Cuffnorm version 2.2.1. We identified a series of genes that were enhanced (>2.0-fold) or reduced (<2.0-fold) for further gene expression analysis. The raw data were deposited in the NCBI Gene Expression Omnibus database under GEO accession number GSE134442. We identified enhanced or suppressed pathways using Qiagen’s Ingenuity Pathway Analysis (IPA; Qiagen Redwood City, www.qiagen.com/ingenuity) with the default settings.

### In vivo tumour growth

The efficiency and safety of miR-4711-5p were assessed in vivo using a tumour xenograft mouse model, as previously described.^19,20^ Cells were mixed with Matrigel (BD Biosciences) and medium at a 1:1 ratio (vol:vol). Approximately 2.5 × 10^6^ cells in 100 μL medium/Matrigel solution were subcutaneously injected into both sides of the lower back regions of 8-week-old female nude mice (NIHON CLEA, Tokyo, Japan). The mice were divided randomly into a parent group (*n* = 4), a negative control group (*n* = 5) and an miR-4711-5p group (*n* = 5) for evaluation of anti-tumour growth effects and safety. Another set of mice were divided randomly into a negative control group (*n* = 3) and an miR-4711-5p group (*n* = 3) to assess the in vivo uptake of miR-4711-5p and its effects on the inhibition of target molecules and the induction of apoptosis. After tumour volumes reached 80 mm^3^, we intravenously administered formulated miRNA with super carbonate apatite (sCA) as the vehicle via the tail vein at a dose of 40 μg per injection.^19,21^ Mice were treated six times with formulated miR-negative control or miR-4711-5p over 2 weeks in the first experiment and treated three consecutive days in the second experiment. Tumour volumes were determined as previously described.^19–22^ The animal facility was SPF and was kept at 20–24 °C. The dark/light cycle was 12/12 h, and cages were plastic, with the maximum number of companions being five adults/cage. All animals could access food and water ad libitum, and food was sterilised. The bedding material had high adsorbing power without dust and was changed every week. At the end of the experiments, all mice were killed by cervical dislocation performed by well-trained individuals. Environmental enrichment was performed with sterile materials. All animal experiments were performed in accordance with currently prescribed guidelines, including the Animal (Scientific Procedures) Act 1986, and following a protocol approved by Osaka University.

### CRC cell cultures from surgically resected clinical samples

We established a spheroid library cultured from surgical specimens resected from patients with CRC at our institute according to a previously reported protocol.^23^ Written informed consent was obtained from all patients, in accordance with the guidelines approved by the Institutional Research Board. This study was conducted under the supervision of the Ethics Board of Osaka University Hospital. For the cell viability test, spheroids were dissociated into single cells by incubation with trypLE express (Invitrogen) for 30 min at 37 °C. Single CRC cells were seeded at a density of 4000 cells per well in 96-well plates. Cell viability was assessed 72 h after transfection of miRNAs.

### Bioinformatics

We used TargetScan (http://www.targetscan.org/) and miRBase (http://www.mirbase.org/) to extract candidate miRNAs targeting KLF5 via the Ingenuity Pathway Analysis MicroRNA Target Filter.

### Statistical analysis

Each experiment was repeated three times. Data are expressed as the mean ± SD. Mean values were compared using Student’s *t*-test. The expression levels of miRNAs in normal colon mucosa and colorectal cancer tissues were analysed using the Wilcoxon signed-rank test. In vivo tumour growth was analysed with one-way ANOVA for repeated measures. Discrete variables were assessed with Fisher’s exact test. *P* values of <0.05 were considered statistically significant. Statistical analyses were performed using JMP Pro 14.0 (SAS Institute, Cary, North Carolina, USA).

## Results

### Screening for candidate miRNAs

**Fig. 1:**
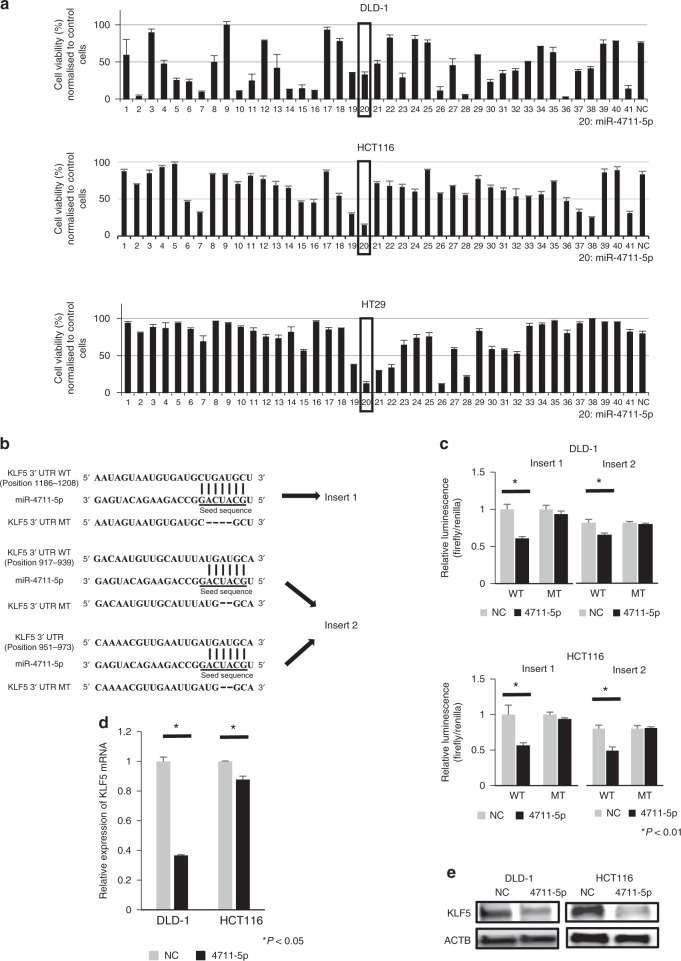
Identification of the KLF5-targeting miRNA miR-4711-5p. **a** Candidate miRNAs were screened by assessing cell viability at 72 h after transfection in CRC cell lines. In three cell lines, miR-4711-5p remarkably suppressed cell viability. Parent cells were used as the control for normalisation. **b** Schematic illustration showing that KLF5 possesses three putative miR-4711-5p binding sites in its 3ʹ-UTR (WT). Four nucleotides in insert 1 and two nucleotides in insert 2 were deleted in the KLF5-mutant plasmids (MT). **c** In CRC cell lines, miR-4711-5p transfection significantly suppressed the luciferase activities of the two reporter plasmids containing three putative miR-4711-5p binding sites in the wild KLF5 3’-UTR (*P* < 0.01) but did not suppress those of KLF5-mutant reporter plasmids. **d**, **e** Real-time quantitative PCR and western blot analyses revealed that miR-4711-5p suppressed KLF5 expression at the mRNA and protein levels (*P* < 0.01). All data represent the mean ± SD.

As a result of in silico analyses by means of miRBase and TargetScan, we identified 41 candidate miRNAs that regulate KLF5 expression. We selected only candidate miRNAs that regulate genes related to at least one of the following three pathways: Wnt/β-catenin signalling, Wnt/Ca^2+^ signalling, and Notch signalling. Of the 44 eligible miRNAs, three were excluded because they were already under assessment at our institute (Supplementary Fig. S2). These candidate miRNAs were transfected into DLD-1, HCT116 and HT29 colon cancer cell lines. The results indicated that miR-4711-5p markedly suppressed cell viability in the three cell lines (Fig. 1a).

### KLF5 is a direct target of miR-4711-5p

In silico analysis showed that KLF5 has three putative miR-4711-5p binding sites in its 3ʹ-UTR (Fig. 1b). Therefore, we constructed two reporter plasmids containing the three putative miR-4711-5p binding sites in the KLF5 3ʹ-UTR. A luciferase reporter assay revealed that miR-4711-5p bound to wild insert 1 and insert 2 and suppressed luciferase activity in DLD-1 and HCT116 cells, whereas a reduction was not observed when plasmids with mismatch sequences were transfected into both cell lines (Fig. 1c). We confirmed that miR-4711-5p suppressed KLF5 expression at the mRNA and protein levels (Fig. 1d, e). These results indicate that KLF5 is a direct target of miR-4711-5p.

### Expression of miR-4711-5p in clinical CRC samples and normal mucosa

When we assessed the expression level of miR-4711-5p in clinical CRC samples from 142 patients, no significant difference was noted between the high and low miR-4711-5p expression groups with regard to overall survival and disease-free survival or the relationship to clinicopathological parameters (Supplementary Fig. S3A, Supplementary Table S3). MiR-4711-5p expression was not significantly different between paired normal mucosa and CRC tissues (*n* = 29) (Supplementary Fig. S3B).

### MiR-4711-5p exhibits anti-tumour effects

**Fig. 2:**
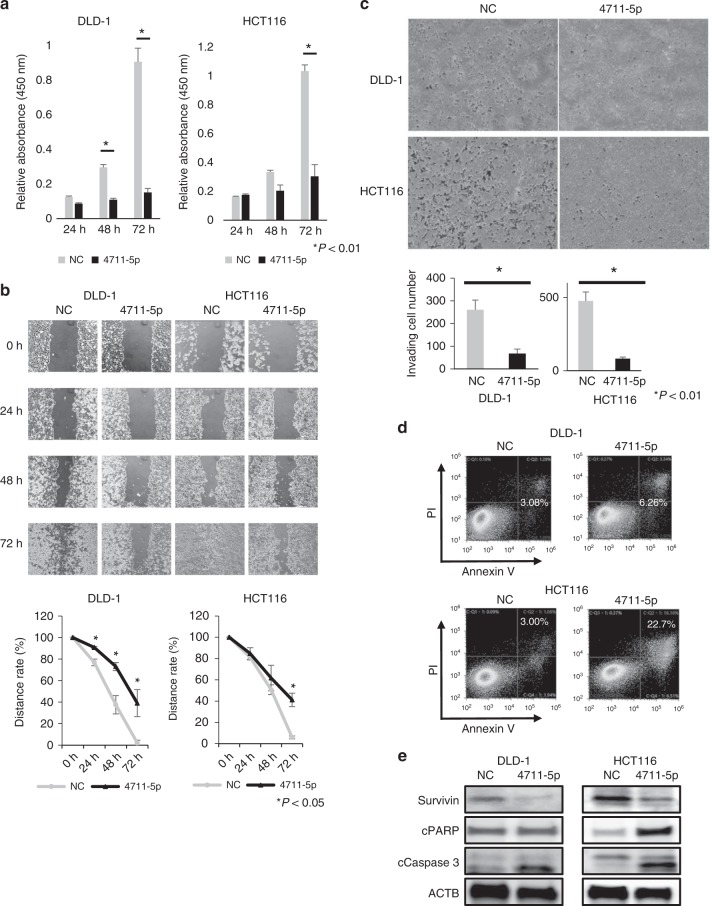
In CRC cell lines, miR-4711-5p exerted various anti-tumour effects. **a** Transfection with miR-4711-5p significantly suppressed the proliferation of DLD-1 and HCT116 cells (*P* < 0.01). **b** In the wound healing assay, miR-4711-5p significantly suppressed the migration ability of DLD-1 and HCT116 cells (*P* < 0.05). **c** Invasion assays revealed that miR-4711-5p significantly suppressed the invasive ability of DLD-1 and HCT116 cells (*P* < 0.01). **d** Annexin V assays showed that miR-4711-5p induced apoptosis, especially in HCT116 cells. **e** Western blotting for apoptosis-associated proteins revealed that miR-4711-5p suppressed survivin expression and increased cleaved caspase 3 expression in both cell lines and increased cleaved PARP expression in HCT116 cells. All data represent the mean ± SD.

MiR-4711-5p significantly suppressed proliferation, migration and invasion abilities in DLD-1 and HCT116 cells compared with the miR-negative control (Fig. 2a–c). We also found that at 48 h after transfection, miR-4711-5p induced a higher rate of apoptosis than the miR-negative control, especially in HCT116 cells (Fig. 2d). Western blotting revealed that miR-4711-5p led to suppression of the anti-apoptotic protein survivin and increased expression of cleaved caspase 3 in both cell lines. Cleaved PARP expression was increased in HCT116 cells treated with miR-4711-5p (Fig. 2e).

### Suppression of stemness of cancer cells

**Fig. 3:**
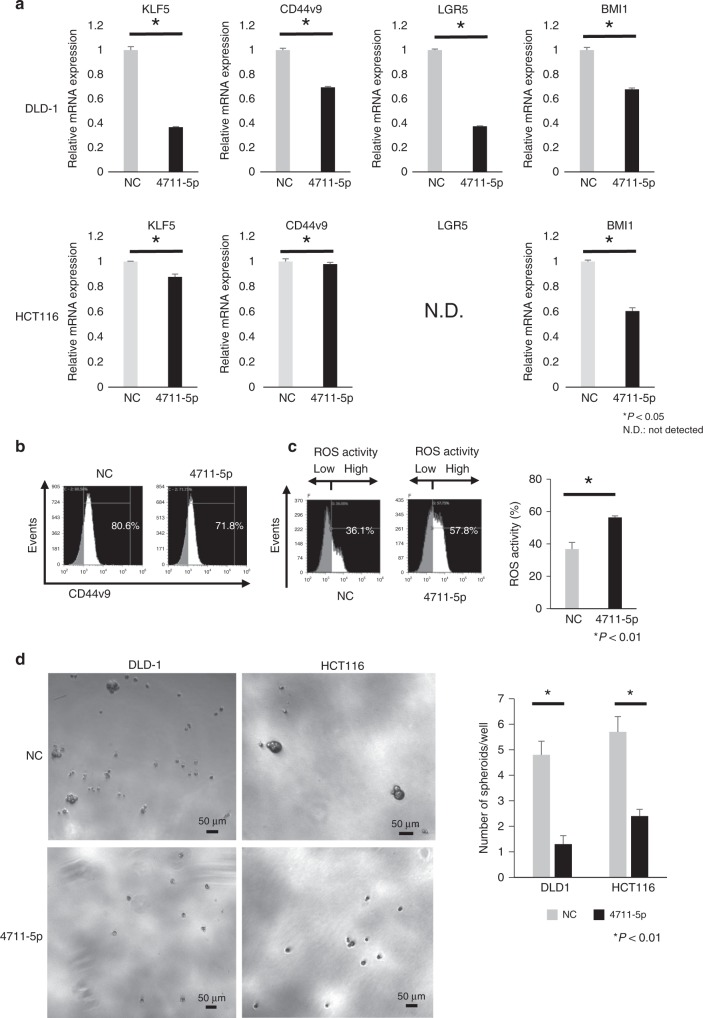
MiR-4711-5p suppressed the stemness of DLD-1 and HCT116 cells. **a** Real-time quantitative PCR revealed that transfection with miR-4711-5p significantly suppressed the expression of putative stem cell markers, such as LGR5, CD44v9 and BMI1, in HCT116 and DLD-1 cells (*P* < 0.05). **b** Flow cytometry analyses of the CSC surface stem cell marker CD44v9 revealed that transfection with miR-4711-5p suppressed CD44v9 expression in DLD-1 cells. **c** Reactive oxygen species (ROS) activity was significantly increased in DLD-1 cells at 24 h after miR-4711-5p transfection (*P* < 0.01). **d** Sphere formation assays revealed that miR-4711-5p transfection significantly decreased the number of spheroids at 1 week after seeding (*P* < 0.01). All data represent the mean ± SD.

We next assessed the change in stemness after transfection of miR-4711-5p and found that miR-4711-5p suppressed the expression of putative stem cell markers (e.g. LGR5, CD44v9 and BMI1) at the mRNA level (Fig. 3a). Additionally, miR-4711-5p suppressed the expression of the CSC surface marker CD44v9 at the protein level (Fig. 3b). ROS activity was enhanced by transfection of miR-4711-5p (Fig. 3c). Sphere formation assays revealed that the number of spheroids was significantly decreased by miR-4711-5p transfection in both DLD-1 and HCT116 cells (Fig. 3d). These findings suggest that miR-4711-5p suppressed the cancer stemness of DLD-1 and HCT116 cells.

### miR-4711-5p provokes G1 arrest in cancer cells

**Fig. 4:**
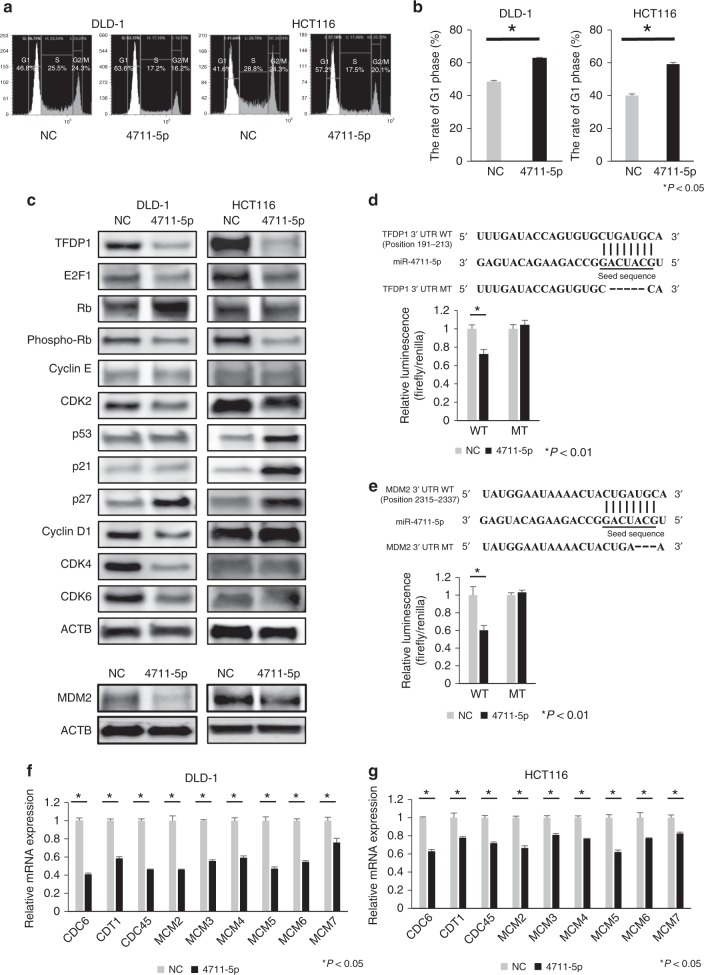
MiR-4711-5p inhibited the G1-to-S phase transition in the cell cycle. **a**, **b** Cell cycle assays revealed that miR-4711-5p significantly increased the ratio of cells in the G1 phase (*P* < 0.01) and decreased the ratio of cells in the S and G2/M phases at 8 h after refeeding with fresh medium supplemented with FBS. **c** Western blotting analyses were performed for cell cycle-related molecules acting at the G1-to-S transition. Proteins, except for MDM2, were extracted at 36 or 48 h after transfection of HCT116 or DLD-1 cells, respectively. MDM2 proteins were extracted at 36 or 12 h after transfection of DLD-1 or HCT116 cells. In both cell lines, miR-4711-5p transfection largely downregulated the expression of TFDP1 and E2F1 and suppressed phosphorylated Rb expression. DLD-1 cells exhibited suppressed expression of CDK2, 4, and 6 and cyclin D1 and upregulated expression of p27^KIP1^. HCT116 cells exhibited increased expression of p21^WAF1/CIP1^ and p27^KIP1^. MDM2 expression was downregulated in both DLD-1 and HCT116 cells. In p53-wild-type HCT116 cells, p53 expression was largely enhanced. **d** Schematic illustration showing the putative miR-4711-5p binding site in the 3ʹ-UTR of TFDP1 (WT). Five nucleotides in the binding site were deleted in the TFDP1-mutant plasmids (MT). In HCT116 cells, miR-4711-5p transfection significantly suppressed the luciferase activities of the reporter plasmids containing the putative miR-4711-5p binding site in the wild-type TFDP1 3’-UTR (*P* < 0.01) but did not suppress those of TFDP1-mutant reporter plasmids. **e** Schematic illustration showing the putative miR-4711-5p binding site in the 3ʹ-UTR of MDM2 (WT). Three nucleotides in the binding site were deleted in the MDM2-mutant plasmids (MT). In HCT116 cells, miR-4711-5p transfection significantly suppressed the luciferase activities of the reporter plasmids containing the putative miR-4711-5p binding site in the wild-type MDM2 3’-UTR (*P* < 0.01) but did not suppress those of MDM2-mutant reporter plasmids. **f**, **g** Real-time quantitative PCR was performed to assess the expression of downstream molecules of the E2F1-TFDP1 heterodimer in DLD-1 (**f**) and HCT116 cells (**g**) transfected with NC or miR-4711-5p. All gene expression levels were significantly downregulated in both cell lines (*P* < 0.05). All data represent the mean ± SD.

Using next-generation sequencing, we assessed the transcriptome changes after transfection of miR-4711-5p. Gene ontology analysis and Ingenuity Pathway Analysis using the RNA sequence data predicted that the cell cycle transition from G1 phase to S phase in DLD-1 cells was inhibited by miR-4711-5p compared to the miR-negative control (Supplementary Fig. S4; Supplementary Table S4). Indeed, cell cycle analyses revealed that miR-4711-5p increased the ratio of cells in the G1 phase and decreased the ratio of cells in the S and G2/M phases in both DLD-1 and HCT116 cells at 8 h after refeeding with fresh medium supplemented with FBS (Fig. 4a, b).

Among cell cycle-related molecules acting at the G1-to-S transition, TFDP1 was identified as a target gene of miR-4711-5p in the TargetScan search. Thus, we focused further investigation on TFDP1, which forms a heterodimer with E2F and regulates genes. A luciferase reporter assay confirmed that miR-4711-5p bound to TFDP1, resulting in the downregulation of TFDP1 protein expression (Fig. 4c, d). In both cell lines, we observed exclusive downregulation of the RNA expression of downstream molecules of E2F-TFDP1, including CDC6, CDT1, MCM7 and others (Fig. 4f, g). We also found that miR-4711-5p transfection resulted in changes in several G1/S checkpoint components in the cell cycle machinery. In DLD-1 cells, CDK2, 4, and 6 and cyclin D1 were suppressed, and the CDK inhibitor p27^KIP1^ was upregulated. In HCT116 cells, p21^WAF1/CIP1^ and p27^KIP1^ were increased. Consequently, the phosphorylation of Rb was suppressed. Notably, p53 expression was largely enhanced in p53-wild-type HCT116 cells, possibly due to downregulation of MDM2, which is a direct target of miR-4711-5p (Fig. 4c, e).

### Anti-cancer effects and safety in vivo

**Fig. 5:**
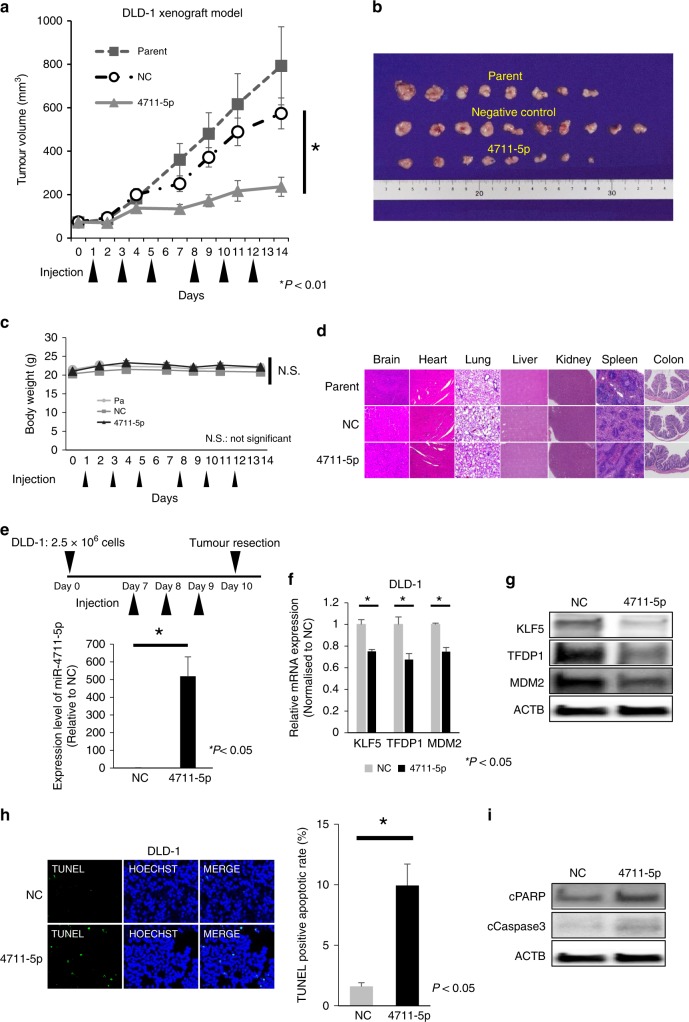
Anti-tumour effects and safety in vivo. Systemic administration of formulated sCA-miR-4711-5p inhibited tumour growth. DLD-1 tumour xenograft mouse models received intravenous administrations of miR-4711-5p or miR-negative control (NC) using sCA as a vehicle on days 0, 2, 4, 7, 9 and 11 via a tail vein (arrows indicate the days of injections). **a**, **b** On day 14, tumours were resected. The results showed that miR-4711-5p significantly inhibited tumour growth in the tumour xenograft mouse model. Tumour volume (A), picture of resected tumours (B). Data represent the mean ± SD. (*P* < 0.01, one-way ANOVA). **c** Body weight did not significantly differ between the groups. **d** Histological findings (H&E staining, × 40) demonstrated that systemic administration of miR-4711-5p had no serious adverse effects on other organ tissues (brain, heart, lung, liver, kidney, spleen and colon). **e, f, g, h**, **i** Nude mice received subcutaneous injections of DLD-1 cells (2 × 10^6^ cells) into the bilateral lower back region. Mice were injected via the tail vein with sCA-formulated miR-4711-5p or miR-NC on days 7, 8 and 9 after cell injection. Tumours were resected on day 10 (**e**, top, *n* = 3 for each group). The expression levels of miR-4711-5p in tumours were measured by qPCR on day 10, and they were significantly upregulated (approximately 500-fold higher than that in the NC-treated group) in tumours of the miR-4711-5p-treated group (**e**, bottom, *P* < 0.05). The expression levels of KLF5, TFDP1 and MDM2 in tumours resected from NC (negative control)-treated and miR-4711-5p-treated mice were assessed at both the mRNA and protein levels (**f, g**). Mir-4711-5p significantly suppressed the expression of KLF5, TFDP1, and MDM2 mRNA compared to NC (*P* < 0.05) (**f**). The protein expression of each target was also downregulated by miR-4711-5p. ACTB bands served as loading controls (**g**). TUNEL assays revealed that miR-4711-5p induced more apoptotic cells than miR-NC (**h**, left). The TUNEL-positive apoptotic cell rate in tumours of the miR-4711-5p-treated group was significantly higher than that in tumours of the miR-NC-treated group (*P* < 0.05) (**h**, right). All data represent the mean ± SD. Western blotting showed that cPARP protein expression was increased by treatment with miR-4711-5p. caspase 3 protein expression was also increased (**i**).

We assessed the anti-tumour effects and safety of miR-4711-5p using a DLD-1 xenograft mouse model. All mice were checked for health status, including microbiological status, before experiments and revealed no abnormalities. Over 2 weeks, mice were injected six times via the tail vein with miR-4711-5p using super carbonate apatite (sCA) as the delivery vehicle. Compared with the miR-negative control and no treatment, miR-4711-5p significantly inhibited tumour growth (Fig. 5a, b). Systemic administration of miR-4711-5p yielded no obvious body weight loss (Fig. 5c). Sections of organ tissues exhibited no particular histological damage by the sCA-miR-4711-5p complex (Fig. 5d). We also confirmed that the subcutaneously established DLD-1 xenograft tumours exhibited approximately 500-fold higher concentrations after three injections of the sCA-miR-4711-5p complex than the xenograft tumours treated with the negative control (day 10, Fig. 5e). In the same tumour sets, the expression levels of target molecules (KLF5, TFDP1 and MDM2) decreased at the mRNA and protein levels (Fig. 5f, g). The TUNEL assay showed that miR-4711-5p induced a significant fraction of apoptotic cells (*P* *<* 0.05, Fig. 5h), and Western blotting showed that cleaved PARP and cleaved caspase 3 expression increased with treatment with miR-4711-5p (Fig. 5i).

### Anti-cancer effects in patient-derived CRC cultured cells

**Fig. 6:**
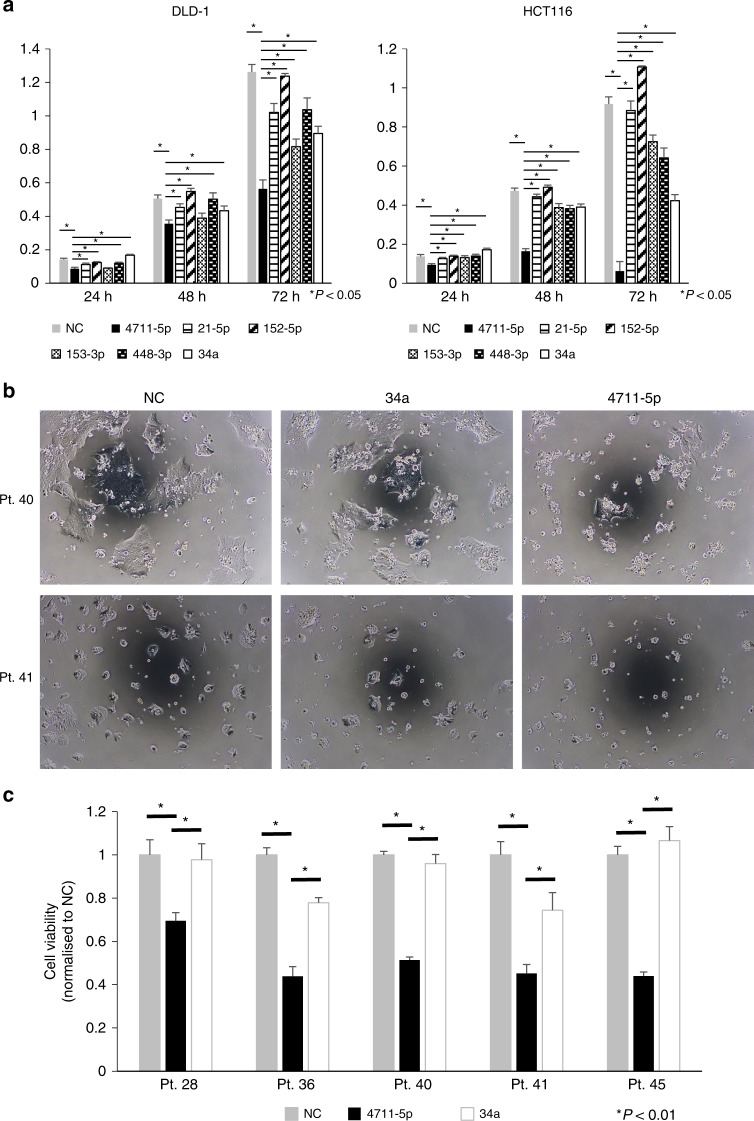
Anti-tumour effects in CRC cell lines and patient-derived tumor cells. **a** Comparison of the tumour growth inhibitory effects of miR-4711-5p and four miRNAs (miR-21, miR-152, miR-153, and miR-448) that have been reported to suppress KLF5 expression. We also included a putative anti-oncomiR, miR-34a, as a positive control. MiR-4711-5p was the most effective in suppressing DLD-1 and HCT116 tumour cell growth among the miRNAs tested (*P* < 0.05). **b**, **c** Comparison of the cell growth inhibitory effects of miR-4711-5p and miR-34a on CRC cells cultured from five different CRC patients (3 stage II and 2 stage III patients). The images of tumour cell cultures derived from two CRC patients (stage II) at 72 h after transfection of miR-NC, miR-34a, and miR-4711-5p (**b**). Cell viability of tumour cells cultured from five different CRC patients at 72 h after transfection. The tumour-suppressive effects of miR-4711-5p were much stronger than those of miR-34a in all patient-derived CRC cell cultures (*P* < 0.01) (**c**). All data represent the mean ± SD.

We compared the inhibitory effect of miR-4711-5p on cell growth with that of the anti-oncomiR miR-34a and previously reported miRNAs that target KLF5, including miR-21-5p, miR-152-5p, miR-153-3p and miR-448-3p.^24–29^ We found that miR-4711-5p exhibited the strongest growth inhibitory effect in DLD-1 and HCT116 cells among the miRNAs tested (Fig. 6a). Next, we assessed the anti-cancer effects of miR-4711-5p using patient-derived CRC cultured cells. The results revealed that miR-4711-5p significantly suppressed the cell viability of CRC cells that were prepared and cultured from five different CRC patients when compared to miR-NC and miR-34a (*P* *<* 0.01, Fig. 6b, c).

## Discussion

In the present study, we demonstrated that miR-4711-5p directly bound to KLF5, induced apoptosis and suppressed cell proliferation, invasion and migration abilities and cancer stemness in CRC cell lines, although apparent cell cycle arrest at the G1-S transition and strong induction of apoptosis, especially in HCT116 cells, may in part have affected the results of the motility assays. We also found that this miRNA provoked G1 arrest through direct inhibition of TFDP1, a heterodimer partner with members of the E2F family. Furthermore, direct binding of miR-4711-5p to MDM2 yielded increased p53 protein expression, leading to induction of apoptosis and G1 arrest in HCT116 cells harbouring wild-type p53. Overall, miR-4711-5p exhibited potent anti-tumour effects in vitro and in vivo, and to our knowledge, this miRNA has not previously been reported.

KLF5 controls the stemness of embryonic stem cells.^11^ Nakaya and colleagues reported that KLF5 facilitated cell proliferation and survival of normal intestinal stem cells and was essential for the oncogenesis of intestinal tumours.^13^ These findings suggest that KLF5 acts as an indispensable stemness-related molecule in both normal and tumour tissues.

The main purpose of our present study was to obtain a therapeutic miRNA that could suppress cancer stemness, and we focused on the KLF5 molecule as the main target. A bioinformatics survey identified 41 candidate miRNAs based on their possibility of suppressing KLF5 expression and other stemness-related signals, such as Wnt/β-catenin signalling and Notch signalling.^30–32^ Of the listed miRNAs, miR-4711-5p exhibited the strongest anti-tumour effects across CRC cell lines and suppressed cancer stemness. Indeed, we further found that this miRNA bound to the 3ʹ-UTR of KLF5 mRNA, leading to downregulation of KLF5 and other CSC markers, including LGR5, BMI1 and CD44v9, as well as inhibiting sphere formation. Since Wnt signalling is associated with cancer stemness, it is of interest that KLF5 can strengthen β-catenin activity by facilitating its nuclear localisation.^13,33,34^

Annexin V assays indicated that apoptosis was induced by miR-4711-5p, especially in HCT116 cells, which harbour wild-type p53. This is not unique because p53 induces apoptosis through various mechanisms, e.g. activation of BAX, NOXA1 and PUMA.^35,36^ It is well known that the p53 protein level is negatively regulated by MDM2-mediated ubiquitination.^37^ Interestingly, we found that miR-4711-5p directly bound to the 3ʹ-UTR of MDM2 mRNA, resulting in decreased MDM2 protein expression. In turn, this caused increased p53 expression in HCT116 cells and induced apoptosis. On the other hand, despite a marked decrease in MDM2 protein, the expression of p53 protein was not affected by miR-4711-5p in DLD-1 cells, which harbour a mutated p53 gene. This result is consistent with prior findings that mutated p53 escapes the degradation-promoting effect of MDM2.^38,39^ Accordingly, apoptosis incidence remained low but was still significantly induced relative to the negative control miRNA treatment. One explanation for apoptosis in this cell type might be the KLF5-dependent regulation of anti-apoptotic survivin. KLF5 has reportedly been shown to bind to the core promoter region of survivin as a transcription factor, thus inducing survivin mRNA expression.^40^ Therefore, it is likely that miR-4711-5p-mediated KLF5 inhibition may lead to the downregulation of survivin (Fig. 2e), which induced apoptosis to some extent.

We performed gene ontology analysis and Ingenuity Pathway Analysis (IPA) using the RNA sequence data, which indicated that miR-4711-5p downregulated genes related to the cell cycle and G1-S checkpoint regulation (Supplementary Table S4; Supplementary Fig. S4). Correspondingly, the cell cycle assay revealed that this miRNA provoked G1 arrest in both cell lines. Among the molecules acting in the machinery of the G1-S transition, we focused on TFDP1 because the TargetScan database indicated that it was a potential direct target of miR-4711-5p and that it forms a heterodimeric complex with E2F1, exerting a crucial role in the progression from G1 to S phase.^41,42^ Indeed, we demonstrated that miR-4711-5p directly bound to the 3ʹ-UTR of TFDP1 mRNA. E2F1 transcriptional activity reportedly regulates the expression of growth-related target genes, such as CDC6, CDT1 and MCM7.^41–43^ Komori et al. demonstrated that TFDP1 knockdown significantly decreased E2F1-dependent activation of the CDC6 promoter.^41^ Likewise, we confirmed that miR-4711-5p exclusively led to downregulation of the gene expression of CDC6, CDT1, CDC45 and MCM family members, all of which are downstream molecules of the TFDP1/E2F1 heterodimer and essential for initiation of DNA replication (ref. ^44^ Supplementary Fig. S5).

We also found that miR-4711-5p induced p27^KIP1^ expression in both cell lines. P27^KIP1^ is a putative CDK inhibitor that binds to the cyclin E-CDK2 complex and cyclin D-CDK4/6 complex and thus negatively regulates the G1-S phase transition.^45–47^ Thus, this activation of p27^KIP1^ could be another reason for the G1 arrest. In the case of HCT116 cells, p53-mediated induction of another CDK inhibitor, p21^WAF1/CIP1^,^36^ could contribute to G1 arrest, which would likely follow downregulation of MDM2 by miR-4711-5p.

According to the miR databases, the expression level of miR-4711-5p in human normal tissues is far lower than that of renowned miRNAs (approximately 1/100th that of miR-34a and 1/1000th that of miR-21) (Supplementary Fig. S6A, B, and C). We found that the expression of miR-4711-5p in CRC tissues was similar to that in their normal counterparts (Supplementary Fig. S3B). As a result, we could not find any correlation between miR-4711-5p expression and various clinical parameters, including patient prognosis. On the other hand, miR-4711-5p exhibited the strongest inhibitory effect on tumour growth when compared with other miRNAs that have been shown to suppress KLF5 in other cancers (i.e. miR-21, miR-152, miR-153 and miR-448^26–29^), as well as a putative anti-oncomiR, miR-34a, which was used in the first clinical trial against human solid tumours.^48,49^ Moreover, administration of miR-4711-5p displayed an outstanding tumour-suppressive effect even compared with miR-34a in five sets of CRC cell cultures that were prepared from CRC patients after surgical resection. These findings indicate that miR-4711-5p is clinically important in terms of cancer therapy, although its usefulness is limited as a biomarker of tumour behaviour. This is, however, not unique because MIRTX, a complementary sequence of miR-29b-1-5p, is not detectable in human tissues, thus having less relevance as a biomarker, whereas it exerts a highly potent tumour-suppressive effect through inhibition of the NFkB signalling pathway by directly binding to the 3’-UTR of CXCR2 and PIK3R1 mRNA.^20,50^

Non-viral nanoparticles are considered safer than viral vectors. Several miRNA-based therapeutics have reached clinical trials, including a mimic of the anti-oncomiR miR-34a for cancer treatment using a liposome as a vehicle.^48,49^ However, the phase I trial of miR-34a was terminated due to immune-related adverse events.^48^ Although several antisense oligonucleotides are in clinical use, miRNA-based therapeutics have not entered clinical application. One of the major challenges is the design of miRNA delivery vehicles that maintain the high stability of the therapeutic miRNAs and transfer them specifically into the targeted tissues, as well as avoiding potential toxicities and off-target effects.^48,51,52^ In this study, we showed that systemic administration of miR-4711-5p mixed with sCA (super carbonate apatite) nanoparticles in nude mice inhibited the growth of pre-established DLD-1 (KRAS^G13D^) xenografts. sCA is a pH-sensitive delivery system for miRNA and siRNA with no significant immune activation.^21^ In earlier studies, we had shown that sCA nanoparticles successfully delivered various nucleic acids or low molecular weight reagents in tumour or IBD (inflammatory bowel disease) model mice with no resulting apparent abnormalities in the body weight, blood chemistry tests or histology of normal organs of the mice.^18–22,53–56^ Moreover, recent review articles have described various miRNA-based therapeutics, most of which employ local injection into tumours, and introduced sCA as a hopeful systemic strategy that could be delivered via intravenous administration.^50,57^ Aiming towards clinical application, we have successfully developed an even less toxic and more efficient delivery system platform that is considered promising in terms of clinical potential.

In conclusion, we identified a promising CSC-targeted therapeutic miRNA: miR-4711-5p. Our data indicate that miR-4711-5p suppresses cancer stemness and exhibits various anti-tumour effects in vitro and significantly suppresses tumour growth in in vivo mouse model without adverse events. MiR-4711-5p delivered by our improved drug delivery system may open a new avenue for miRNA-based therapeutics against cancers in the near future.

## Supplementary information

Supplementary Figures and Tables

## Data Availability

The expression levels of miR-4711-5p and other miRNAs referred to in this study (Supplementary Fig. S6) are available on the Tissue Atlas website (https://ccb-web.cs.uni-saarland.de/tissueatlas/). The expression levels of KLF5 referred to in this study (Supplementary Fig. S1) are available on the Human Protein Atlas website (https://www.proteinatlas.org/). All the other data of the study can be found with the corresponding author.
