# Supplementary material for: miR-4711-5p regulates cancer stemness and cell cycle progression via KLF5, MDM2 and TFDP1 in colon cancer cells
Source: Br J Cancer. 2020 Feb 18;122(7):1037–49. doi: 10.1038/s41416-020-0758-1 (PMC7109136; doi:10.1038/s41416-020-0758-1)
Supplement: Supplementary file 1 — Supplementary Figures and Tables [file 41416_2020_758_MOESM1_ESM.pptx]

## Slide 1
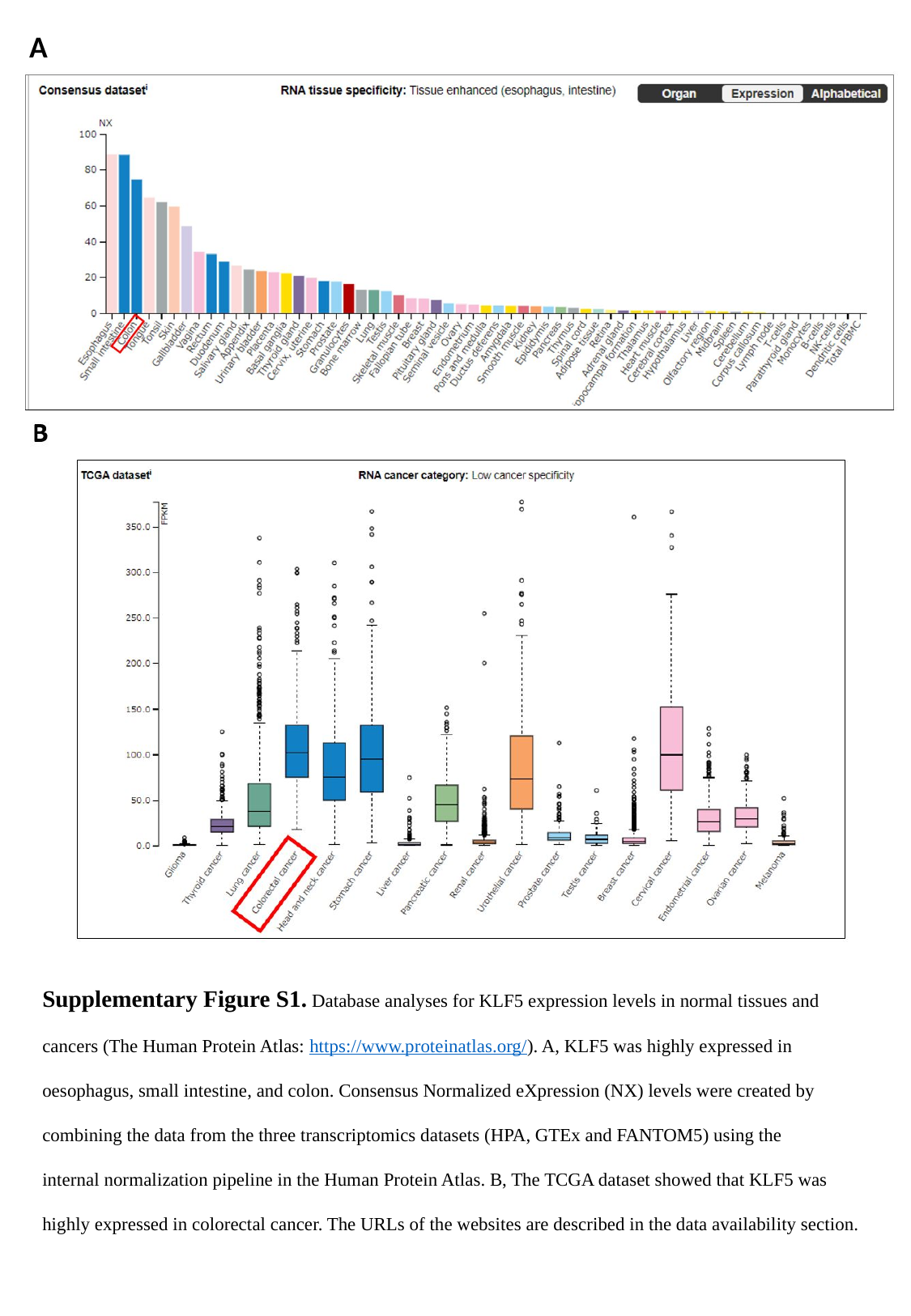

A
Supplementary Figure S1. Database analyses for KLF5 expression levels in normal tissues and cancers (The Human Protein Atlas: https://www.proteinatlas.org/). A, KLF5 was highly expressed in oesophagus, small intestine, and colon. Consensus Normalized eXpression (NX) levels were created by combining the data from the three transcriptomics datasets (HPA, GTEx and FANTOM5) using the internal normalization pipeline in the Human Protein Atlas. B, The TCGA dataset showed that KLF5 was highly expressed in colorectal cancer. The URLs of the websites are described in the data availability section.

## Slide 2
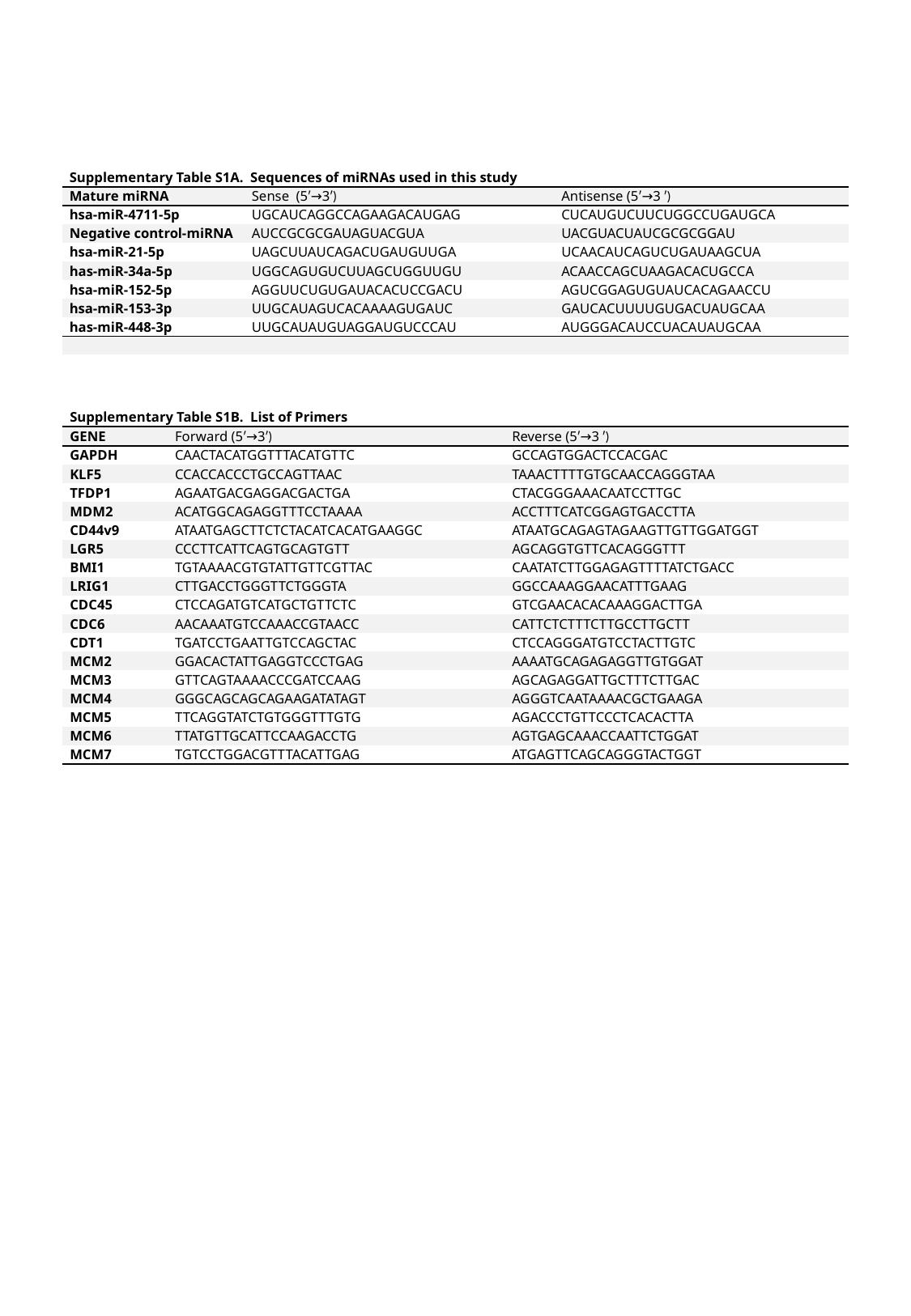

| Supplementary Table S1A. Sequences of miRNAs used in this study | | |
| --- | --- | --- |
| Mature miRNA | Sense (5ʹ→3ʹ) | Antisense (5ʹ→3 ʹ) |
| hsa-miR-4711-5p | UGCAUCAGGCCAGAAGACAUGAG | CUCAUGUCUUCUGGCCUGAUGCA |
| Negative control-miRNA | AUCCGCGCGAUAGUACGUA | UACGUACUAUCGCGCGGAU |
| hsa-miR-21-5p | UAGCUUAUCAGACUGAUGUUGA | UCAACAUCAGUCUGAUAAGCUA |
| has-miR-34a-5p | UGGCAGUGUCUUAGCUGGUUGU | ACAACCAGCUAAGACACUGCCA |
| hsa-miR-152-5p | AGGUUCUGUGAUACACUCCGACU | AGUCGGAGUGUAUCACAGAACCU |
| hsa-miR-153-3p | UUGCAUAGUCACAAAAGUGAUC | GAUCACUUUUGUGACUAUGCAA |
| has-miR-448-3p | UUGCAUAUGUAGGAUGUCCCAU | AUGGGACAUCCUACAUAUGCAA |
| | | |
| Supplementary Table S1B. List of Primers | | |
| --- | --- | --- |
| GENE | Forward (5ʹ→3ʹ) | Reverse (5ʹ→3 ʹ) |
| GAPDH | CAACTACATGGTTTACATGTTC | GCCAGTGGACTCCACGAC |
| KLF5 | CCACCACCCTGCCAGTTAAC | TAAACTTTTGTGCAACCAGGGTAA |
| TFDP1 | AGAATGACGAGGACGACTGA | CTACGGGAAACAATCCTTGC |
| MDM2 | ACATGGCAGAGGTTTCCTAAAA | ACCTTTCATCGGAGTGACCTTA |
| CD44v9 | ATAATGAGCTTCTCTACATCACATGAAGGC | ATAATGCAGAGTAGAAGTTGTTGGATGGT |
| LGR5 | CCCTTCATTCAGTGCAGTGTT | AGCAGGTGTTCACAGGGTTT |
| BMI1 | TGTAAAACGTGTATTGTTCGTTAC | CAATATCTTGGAGAGTTTTATCTGACC |
| LRIG1 | CTTGACCTGGGTTCTGGGTA | GGCCAAAGGAACATTTGAAG |
| CDC45 | CTCCAGATGTCATGCTGTTCTC | GTCGAACACACAAAGGACTTGA |
| CDC6 | AACAAATGTCCAAACCGTAACC | CATTCTCTTTCTTGCCTTGCTT |
| CDT1 | TGATCCTGAATTGTCCAGCTAC | CTCCAGGGATGTCCTACTTGTC |
| MCM2 | GGACACTATTGAGGTCCCTGAG | AAAATGCAGAGAGGTTGTGGAT |
| MCM3 | GTTCAGTAAAACCCGATCCAAG | AGCAGAGGATTGCTTTCTTGAC |
| MCM4 | GGGCAGCAGCAGAAGATATAGT | AGGGTCAATAAAACGCTGAAGA |
| MCM5 | TTCAGGTATCTGTGGGTTTGTG | AGACCCTGTTCCCTCACACTTA |
| MCM6 | TTATGTTGCATTCCAAGACCTG | AGTGAGCAAACCAATTCTGGAT |
| MCM7 | TGTCCTGGACGTTTACATTGAG | ATGAGTTCAGCAGGGTACTGGT |

## Slide 3
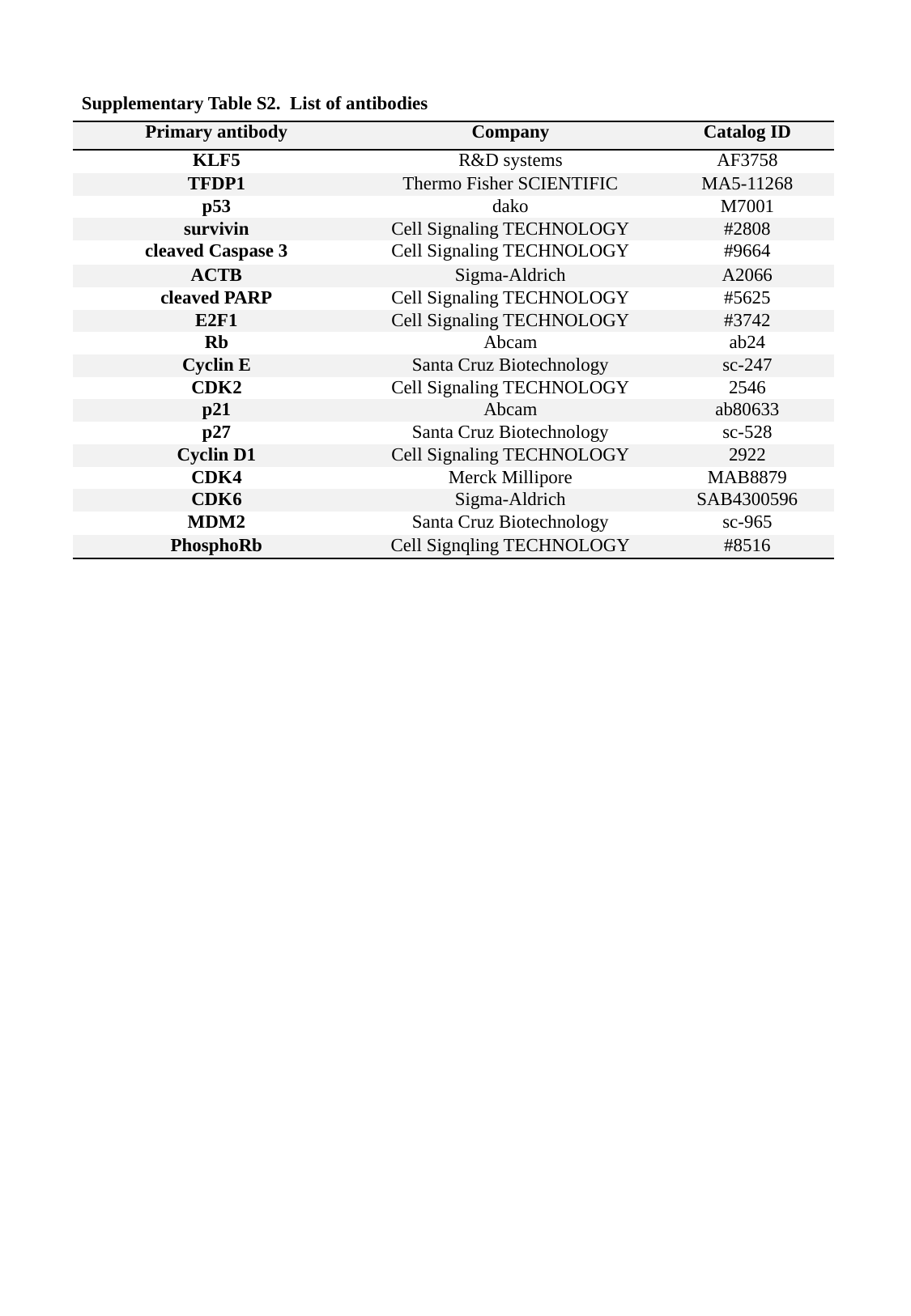

| Supplementary Table S2. List of antibodies | | |
| --- | --- | --- |
| Primary antibody | Company | Catalog ID |
| KLF5 | R&D systems | AF3758 |
| TFDP1 | Thermo Fisher SCIENTIFIC | MA5-11268 |
| p53 | dako | M7001 |
| survivin | Cell Signaling TECHNOLOGY | #2808 |
| cleaved Caspase 3 | Cell Signaling TECHNOLOGY | #9664 |
| ACTB | Sigma-Aldrich | A2066 |
| cleaved PARP | Cell Signaling TECHNOLOGY | #5625 |
| E2F1 | Cell Signaling TECHNOLOGY | #3742 |
| Rb | Abcam | ab24 |
| Cyclin E | Santa Cruz Biotechnology | sc-247 |
| CDK2 | Cell Signaling TECHNOLOGY | 2546 |
| p21 | Abcam | ab80633 |
| p27 | Santa Cruz Biotechnology | sc-528 |
| Cyclin D1 | Cell Signaling TECHNOLOGY | 2922 |
| CDK4 | Merck Millipore | MAB8879 |
| CDK6 | Sigma-Aldrich | SAB4300596 |
| MDM2 | Santa Cruz Biotechnology | sc-965 |
| PhosphoRb | Cell Signqling TECHNOLOGY | #8516 |

## Slide 4
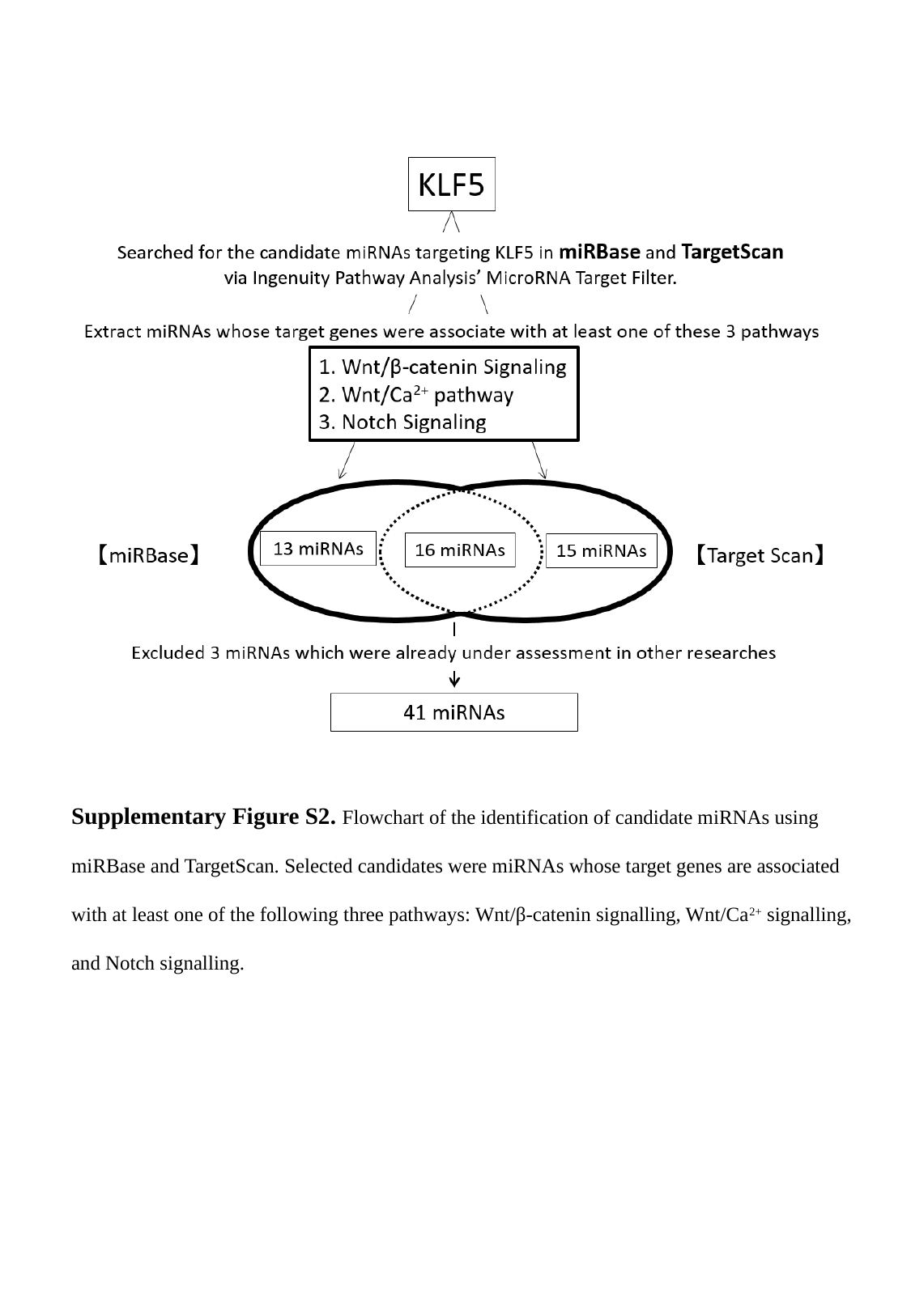

Supplementary Figure S2. Flowchart of the identification of candidate miRNAs using miRBase and TargetScan. Selected candidates were miRNAs whose target genes are associated with at least one of the following three pathways: Wnt/β-catenin signalling, Wnt/Ca2+ signalling, and Notch signalling.

## Slide 5
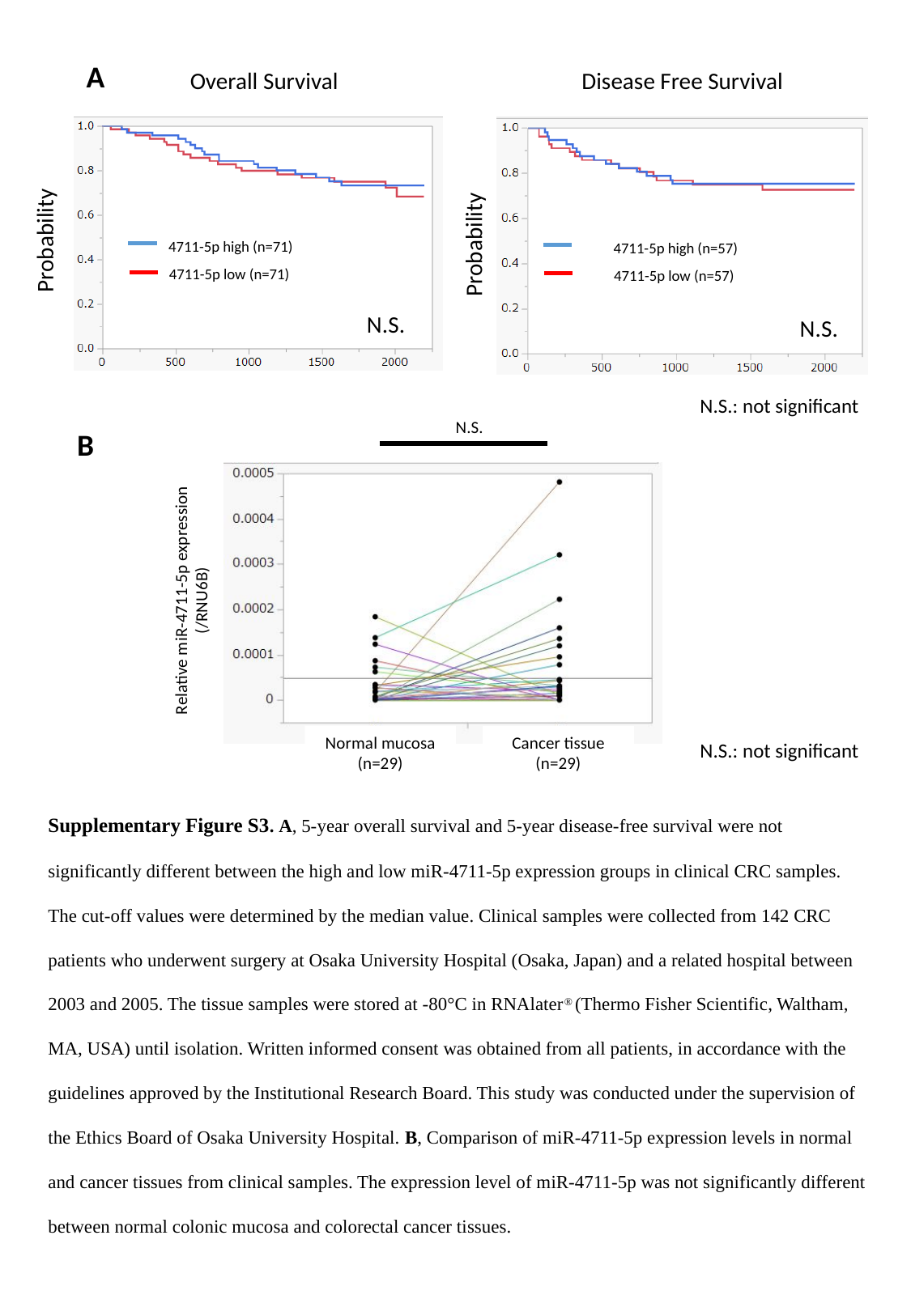

A
Overall Survival
Disease Free Survival
Probability
Probability
4711-5p high (n=71)
4711-5p high (n=57)
4711-5p low (n=71)
4711-5p low (n=57)
 N.S.
 N.S.
 N.S.: not significant
N.S.
B
Relative miR-4711-5p expression
(/RNU6B)
Normal mucosa (n=29)
Cancer tissue (n=29)
 N.S.: not significant
Supplementary Figure S3. A, 5-year overall survival and 5-year disease-free survival were not significantly different between the high and low miR-4711-5p expression groups in clinical CRC samples. The cut-off values were determined by the median value. Clinical samples were collected from 142 CRC patients who underwent surgery at Osaka University Hospital (Osaka, Japan) and a related hospital between 2003 and 2005. The tissue samples were stored at -80°C in RNAlater® (Thermo Fisher Scientific, Waltham, MA, USA) until isolation. Written informed consent was obtained from all patients, in accordance with the guidelines approved by the Institutional Research Board. This study was conducted under the supervision of the Ethics Board of Osaka University Hospital. B, Comparison of miR-4711-5p expression levels in normal and cancer tissues from clinical samples. The expression level of miR-4711-5p was not significantly different between normal colonic mucosa and colorectal cancer tissues.

## Slide 6
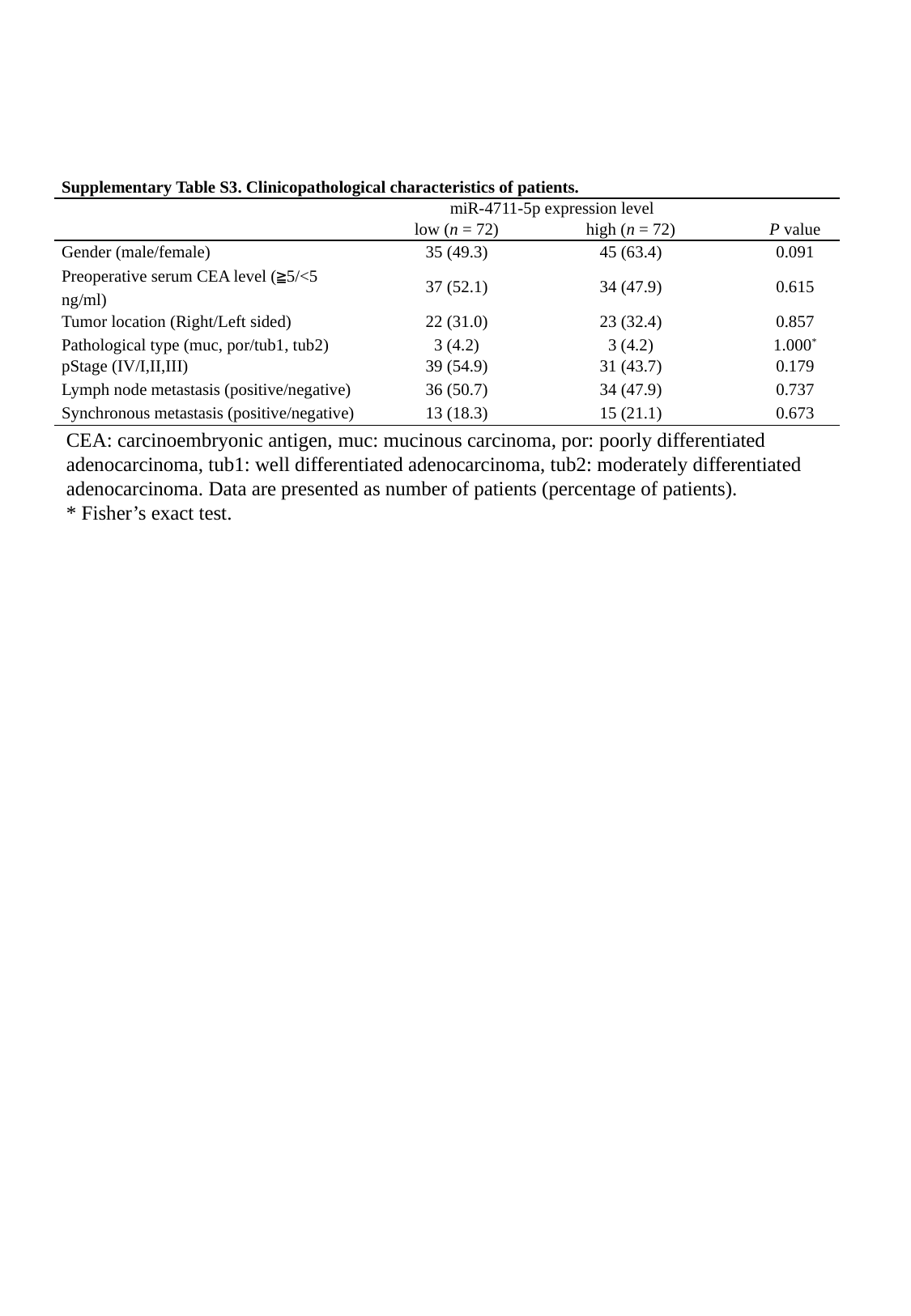

| Supplementary Table S3. Clinicopathological characteristics of patients. | | | | | |
| --- | --- | --- | --- | --- | --- |
| | | | miR-4711-5p expression level | | |
| | | | low (n = 72) | high (n = 72) | P value |
| Gender (male/female) | | | 35 (49.3) | 45 (63.4) | 0.091 |
| Preoperative serum CEA level (≧5/<5 ng/ml) | | | 37 (52.1) | 34 (47.9) | 0.615 |
| Tumor location (Right/Left sided) | | | 22 (31.0) | 23 (32.4) | 0.857 |
| Pathological type (muc, por/tub1, tub2) | | | 3 (4.2) | 3 (4.2) | 1.000\* |
| pStage (IV/I,II,III) | | | 39 (54.9) | 31 (43.7) | 0.179 |
| Lymph node metastasis (positive/negative) | | | 36 (50.7) | 34 (47.9) | 0.737 |
| Synchronous metastasis (positive/negative) | | | 13 (18.3) | 15 (21.1) | 0.673 |
CEA: carcinoembryonic antigen, muc: mucinous carcinoma, por: poorly differentiated adenocarcinoma, tub1: well differentiated adenocarcinoma, tub2: moderately differentiated adenocarcinoma. Data are presented as number of patients (percentage of patients).
* Fisher’s exact test.

## Slide 7
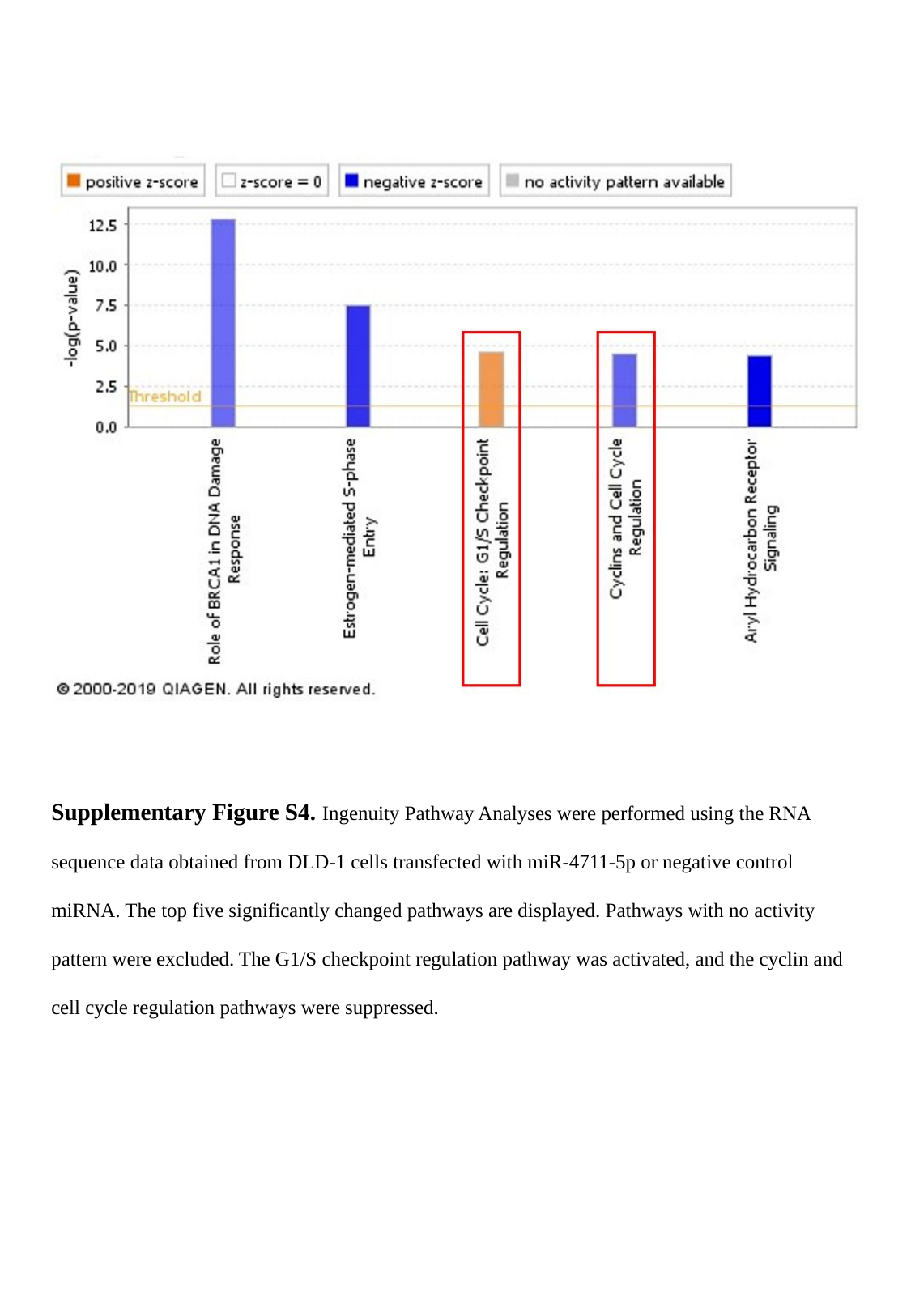

Supplementary Figure S4. Ingenuity Pathway Analyses were performed using the RNA sequence data obtained from DLD-1 cells transfected with miR-4711-5p or negative control miRNA. The top five significantly changed pathways are displayed. Pathways with no activity pattern were excluded. The G1/S checkpoint regulation pathway was activated, and the cyclin and cell cycle regulation pathways were suppressed.

## Slide 8
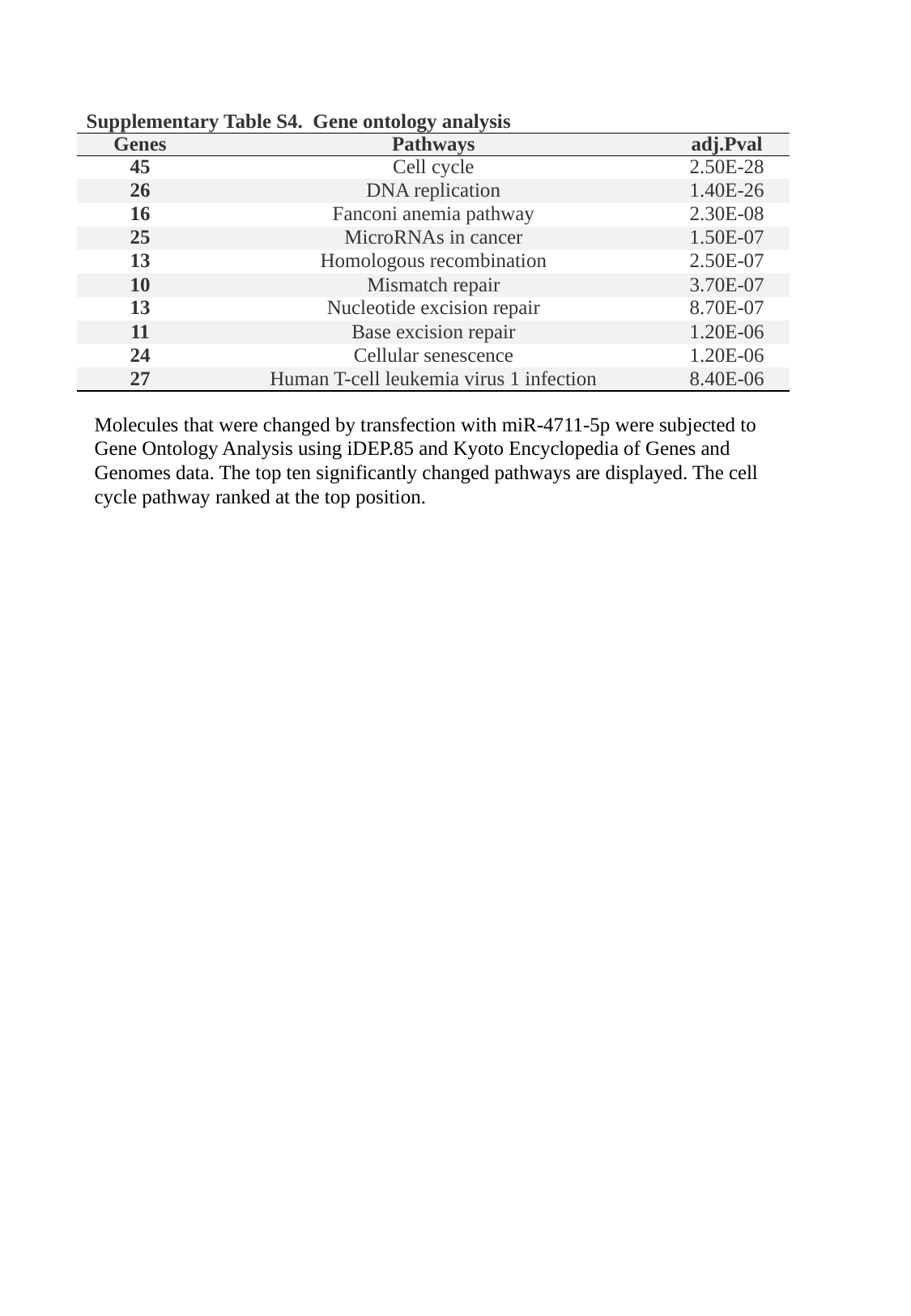

| Supplementary Table S4. Gene ontology analysis | | |
| --- | --- | --- |
| Genes | Pathways | adj.Pval |
| 45 | Cell cycle | 2.50E-28 |
| 26 | DNA replication | 1.40E-26 |
| 16 | Fanconi anemia pathway | 2.30E-08 |
| 25 | MicroRNAs in cancer | 1.50E-07 |
| 13 | Homologous recombination | 2.50E-07 |
| 10 | Mismatch repair | 3.70E-07 |
| 13 | Nucleotide excision repair | 8.70E-07 |
| 11 | Base excision repair | 1.20E-06 |
| 24 | Cellular senescence | 1.20E-06 |
| 27 | Human T-cell leukemia virus 1 infection | 8.40E-06 |
Molecules that were changed by transfection with miR-4711-5p were subjected to Gene Ontology Analysis using iDEP.85 and Kyoto Encyclopedia of Genes and Genomes data. The top ten significantly changed pathways are displayed. The cell cycle pathway ranked at the top position.

## Slide 9
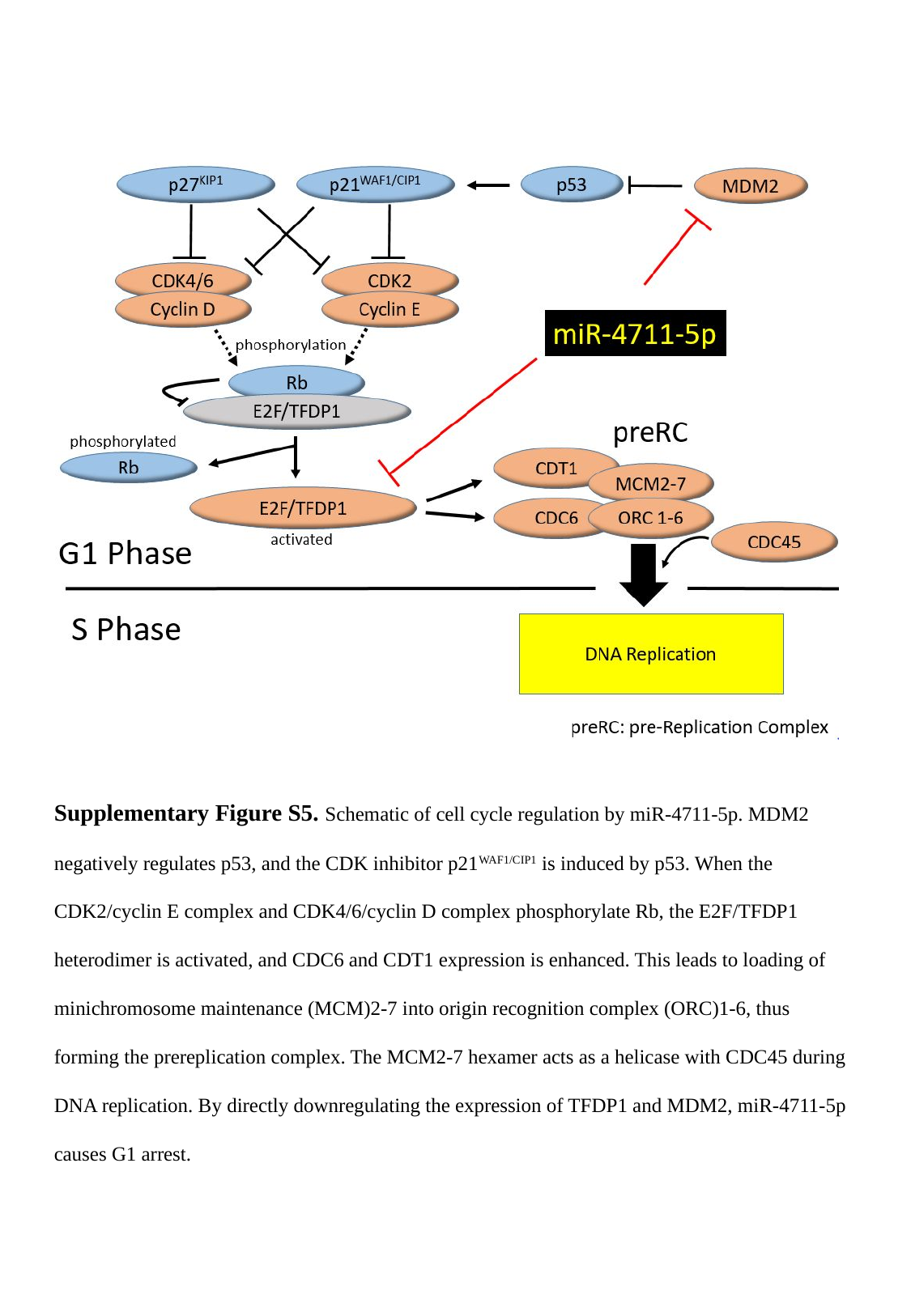

Supplementary Figure S5. Schematic of cell cycle regulation by miR-4711-5p. MDM2 negatively regulates p53, and the CDK inhibitor p21WAF1/CIP1 is induced by p53. When the CDK2/cyclin E complex and CDK4/6/cyclin D complex phosphorylate Rb, the E2F/TFDP1 heterodimer is activated, and CDC6 and CDT1 expression is enhanced. This leads to loading of minichromosome maintenance (MCM)2-7 into origin recognition complex (ORC)1-6, thus forming the prereplication complex. The MCM2-7 hexamer acts as a helicase with CDC45 during DNA replication. By directly downregulating the expression of TFDP1 and MDM2, miR-4711-5p causes G1 arrest.

## Slide 10
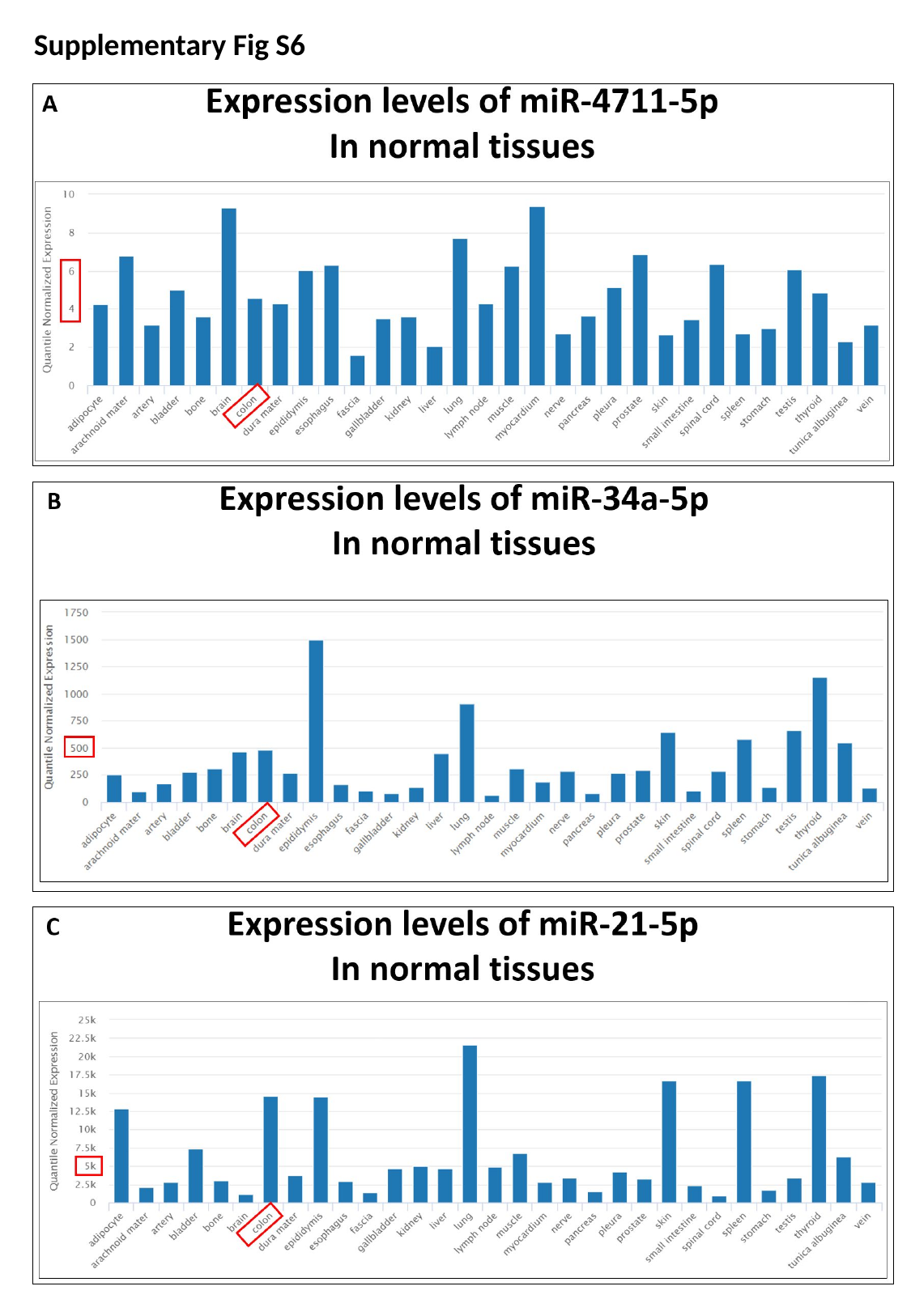

Supplementary Fig S6

## Slide 11
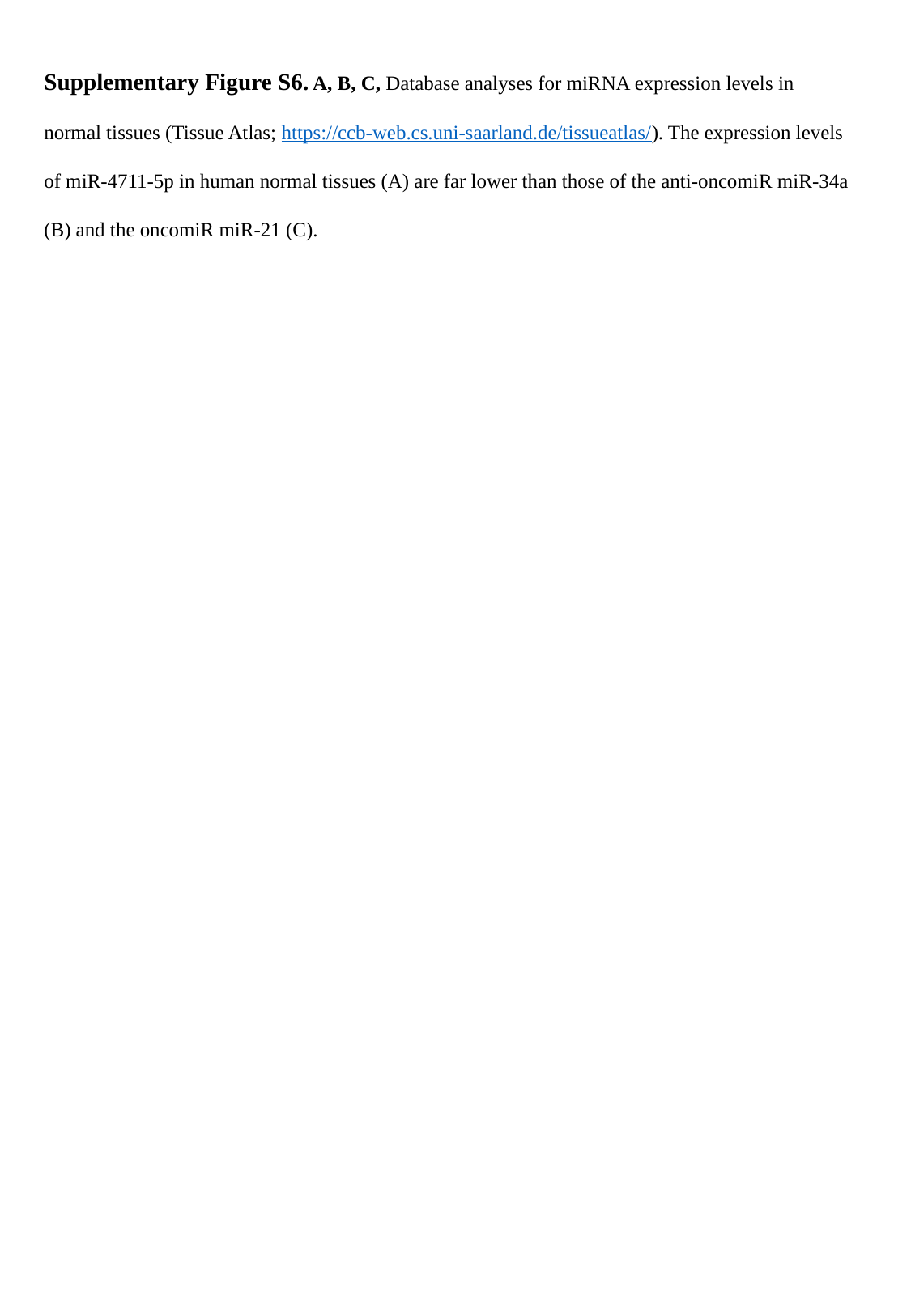

Supplementary Figure S6. A, B, C, Database analyses for miRNA expression levels in normal tissues (Tissue Atlas; https://ccb-web.cs.uni-saarland.de/tissueatlas/). The expression levels of miR-4711-5p in human normal tissues (A) are far lower than those of the anti-oncomiR miR-34a (B) and the oncomiR miR-21 (C).
